# Regulation of human glioblastoma cell death by combined treatment of cannabidiol, γ-radiation and small molecule inhibitors of cell signaling pathways

**DOI:** 10.18632/oncotarget.18240

**Published:** 2017-05-27

**Authors:** Vladimir N. Ivanov, Jinhua Wu, Tom K. Hei

**Affiliations:** ^1^ Center for Radiological Research, Department of Radiation Oncology, College of Physicians and Surgeons, Columbia University, New York, NY 10032, USA

**Keywords:** glioblastoma, cannabidiol, γ-radiation, apoptosis, MAPK p38

## Abstract

Glioblastoma (GBM) is the most common primary malignant brain tumor in adults. The challenging problem in cancer treatment is to find a way to upregulate radiosensitivity of GBM while protecting neurons and neural stem/progenitor cells in the brain. The goal of the present study was upregulation of the cytotoxic effect of γ-irradiation in GBM by non-psychotropic and non-toxic cannabinoid, cannabidiol (CBD). We emphasized three main aspects of signaling mechanisms induced by CBD treatment (alone or in combination with γ-irradiation) in human GBM that govern cell death: 1) CBD significantly upregulated the active (phosphorylated) JNK1/2 and MAPK p38 levels with the subsequent downregulation of the active phospho-ERK1/2 and phospho-AKT1 levels. MAPK p38 was one of the main drivers of CBD-induced cell death, while death levels after combined treatment of CBD and radiation were dependent on both MAPK p38 and JNK. Both MAPK p38 and JNK regulate the endogenous TRAIL expression. 2) NF-κB p65-P(Ser536) was not the main target of CBD treatment and this transcription factor was found at high levels in CBD-treated GBM cells. Additional suppression of p65-P(Ser536) levels using specific small molecule inhibitors significantly increased CBD-induced apoptosis. 3) CBD treatment substantially upregulated TNF/TNFR1 and TRAIL/TRAIL-R2 signaling by modulation of both ligand and receptor levels followed by apoptosis. Our results demonstrate that radiation-induced death in GBM could be enhanced by CBD-mediated signaling in concert with its marginal effects for neural stem/progenitor cells and astrocytes. It will allow selecting efficient targets for sensitization of GBM and overcoming cancer therapy-induced severe adverse sequelae.

## INTRODUCTION

External beam radiation therapy alone or in combination with chemotherapy (using Temozolomide, a DNA methylating agent with DNA damage activity) is the main treatment procedure for brain tumors including glioblastoma [1, 2]. Such treatment also causes severe damage to normal brain tissues. While normal glial cells exhibit a substantial degree of radioresistance, adult neurons and endothelial cells could be significantly injured by ionizing radiation. Additionally, oligodendrocyte precursor cells (OPC), neural stem and progenitor cells (NSC/NPC) having significant proliferative capacities are also highly sensitive to radiation therapy alone or especially in combination with chemotherapy. Clinical observations and experiments with animals further demonstrated that cranial irradiation for brain tumor treatment may result in encephalopathy, as well as strong cognitive and memory deficits [3–10].

Glioblastoma (GBM) is the most common primary malignant brain tumor in adults affecting more than 23,000 new patients in the US each year with estimated number of death 16,050 in the year 2016 (http://seer.cancer.gov/statfacts/html/brain.html#risk). Despite advances in therapy, outcomes remain poor with a median survival rate of 12–15 months after initial diagnosis [11]. The challenging problem in brain cancer therapy is to find a way to radiosensitize GBM while protecting neurons, NSC/NPC and OPC or elucidate new ways of treatment.

Gliomas are thought to arise from NSC/NPC and young astrocytes [12]. Numerous progression-associated genetic alterations are very common to different types of gliomas and glioblastomas. Investigations of the somatic genomic landscape of GBM’s demonstrated a connection between gene alteration and signaling pathway modifications in GBM cells, which include receptor tyrosine kinases (RTK) pathways (EGFR1, FGFR2 or PDGFRA), PI3K/PTEN-AKT-mTORC1 pathway, MAPK pathways, p53 pathway, RB1 pathway (RB1, CDK4 and CDK6) [9, 13] and IKK-NF-κB pathway with frequent inactivation of inhibitor kappa-B (*NFKBIA*) [14, 15]. These genetic alterations together with numerous epigenetic modifications could establish resistance to treatment of GBM. Furthermore, quick rewiring of signaling networks that substantially changes gene expression in GBM cells and provide resistance to treatment may occur through “adaptive” mechanisms before clonal selection in the heterogeneous GBM cell populations [16].

Treatment with ionizing radiation induces the generation of reactive oxygen species (ROS), which affect genomic DNA in the nucleus (resulting in DNA damage followed by a DNA damage response) and initiate cell signaling pathways in the cytoplasm that control gene expression in the regulation of cell survival/death [17, 18]. The tumor microenvironment is actively involved in the control of cell signaling pathways and gene expression in cancer cells before and after treatment by irradiation using intercellular communications [19]. On the other hand, beside direct lethal effects on target cells, γ-radiation induced expression and secretion of cytokines, death ligands, prostaglandins and endocannabinoids establishing inverse communication between treated tumor cells and non-irradiated bystander cells of the microenvironment, as well as of remote regions in the brain containing OPC and NSC/NPC [20–22]. These secondary non-targeted effects could significantly affect normal cells and upregulate direct destructive effects of radiation [20, 21, 23].

Human GBM’s are extremely heterogeneous at both levels of regulation during carcinogenesis: i) genomic alterations, including chromosome rearrangement, deletions, and mutations; ii) substantial epigenetic changes that control gene expression. The integrated genomic analysis built a dataset from 200 GBM’s and normal brain samples allowed to identify four glioblastoma subtypes named proneural, classical, mesenchymal and neural each characterized by a distinct gene expression signature [24]. i) The proneural subtype was associated with Platelet-derived growth factor receptor (PDGFRA) abnormalities, *IDH1* and *TP53* mutations. ii) The classical subtype was strongly associated with the astrocytic signature and contained all common genomic aberrations observed in GBM, such as chromosome 7 amplifications, chromosome 10 deletions, *EGFR* amplification, deletion of the TP53-stabilising isoform of the cyclin-dependent inhibitor *CDKN2A/ARF*. iii) The mesenchymal subtype was characterized by *NF1* abnormalities quite often together with *PTEN* mutations/deletions. Furthermore, genes in the TNF superfamily and NF-κB pathway were highly expressed in this subtype together with the expression of astrocyte and mesenchymal markers. It was the most aggressive subtype with the poor outcome of patients. iv) The neural subtype was typified by expression of neuron markers with relatively low levels of mutated driver genes, such as *TP53, PTEN, NF1, EGFR1* and *IDH*1 [24].

Three human glioblastoma cell lines, U87MG, U118MG, and T98G used in the current study contain numerous rearrangements of chromosomes and substantial changes in the genomic landscape. However, they represent poorly subtypes of primary human gliomas. On the other hand, U87MG and U118MG were used as parent lines for selections of sublines of typical mesenchymal pattern cells [25, 26]. In spite of obvious restriction of similarity between glioblastoma cells *in vivo* and in cell culture conditions, a recent comprehensive study highlighted the importance of established cell lines that represent the same pattern of gene alteration as cancer cells *in vivo* [27]. In the present study, we elucidate the killing effects and mechanisms of sensitization of GBM cells to treatment through signaling pathways induced by the exogenous cannabinoids that could regulate the signaling cascades initiating death of cancer cells [28, 29]. Numerous investigations of the last decade demonstrated cytotoxic effects of cannabinoids, including non-toxic cannabidiol (CBD) without psychogenic activity, on human and mouse glioblastoma cells [29–33]. However, the signaling mechanisms that are involved in regulation of glioblastoma cell death and survival by CBD are still not completely elucidated. There is interest to investigate possible radiosensitization of human GBM cells by combined treatment of CBD and γ-irradiation with further use of specific inhibitors of the distinct signaling pathways that could enhance or suppress cell death.

The endocannabinoid system regulates general and neuro-specific function through cannabinoid receptor-1 (CB1), which is preferentially expressed in neurons but also in other types of cells, and cannabinoid receptor-2 (CB2), which is preferentially expressed on lymphocytes, as well as in many other cells. Glial cells and gliomas possess both CB receptors [34, 35]. Endocannabinoids and ∆^9^-tetrahydrocannabinol ∆THC have a high affinity for both cannabinoid receptors, CB1 and CB2, which are members of the superfamily of Seven-transmembrane-domain G-protein-coupled receptors that induce upon activation signaling cascades in the cells. However, due to the very low affinity of CBD for both CB1 and CB2, CBD-induced signaling effects in GBM cells were suggested to be mostly CB1/2-receptor-independent [30, 32]. In spite of this feature, a downstream cross-talk between CBD-mediated signaling and CB1- and CB2-dependent signaling cascades might occur in an indirect manner using an unknown mechanism [36, 37]. In contrast to relatively normal functions in neuronal and glial cells, the early effects of ∆THC-activated CB1/2 receptors in glioma/glioblastoma cells included a substantial upregulation of ceramide levels in the endoplasmic reticulum (ER) that resulted in the ER-stress response followed by autophagy and apoptosis [38, 39]. On the other hand, CBD treatment induced massive ROS production accompanied by activation of both ROS-dependent signaling and the protective antioxidant systems in glioma cells linked with the subsequent induction of autophagy and activation of the mitochondrial apoptotic pathway [40–42]. CBD can also induce cancer cell apoptosis via activation of p53-dependent apoptotic pathways in cancer cells with wild-type p53 [43]. In contrast, CBD treatment of nonmalignant brain cells was not linked with induction of apoptosis [44].

Combined treatment of brain cancers could be a way to increase radiosensitivity of GBM while protecting neurons and NSC/NPC. The main goal of the present study was to investigate enhanced cytotoxic effects of γ-irradiation in GBM by non-psychotropic and non-toxic cannabinoid, cannabidiol (CBD) and to elucidate cell signaling pathways that mediate these effects.

## RESULTS

### Combined treatment of glioblastoma cells by cannabidiol (CBD) and γ-irradiation

Treatment of GBM cells with high doses of γ-radiation suppressed cell proliferation and induced a mixed type of cell death mainly through cell cycle arrest, mitotic catastrophe, apoptosis, and secondary necrosis. Temozolomide, a DNA methylating agent with a strong DNA damage activity further amplified these processes [2, 17, 45]. As an alternative modality to increase the lethal effects of γ-irradiation, we co-treated human glioblastoma (GBM) cells with cannabidiol (CBD, 5–20 µM) suggesting strong additional effects on regulation of cell signaling, proliferation, and death. Three human glioblastoma lines, U87MG (HTB-14), U118MG (HTB-15), and T98G (CRL-1690) used in the present study were obtained from the ATCC. These lines exhibit high protein expression of cannabinoid receptors, CB1 and CB2 [34, 35].

**Figure 1:**
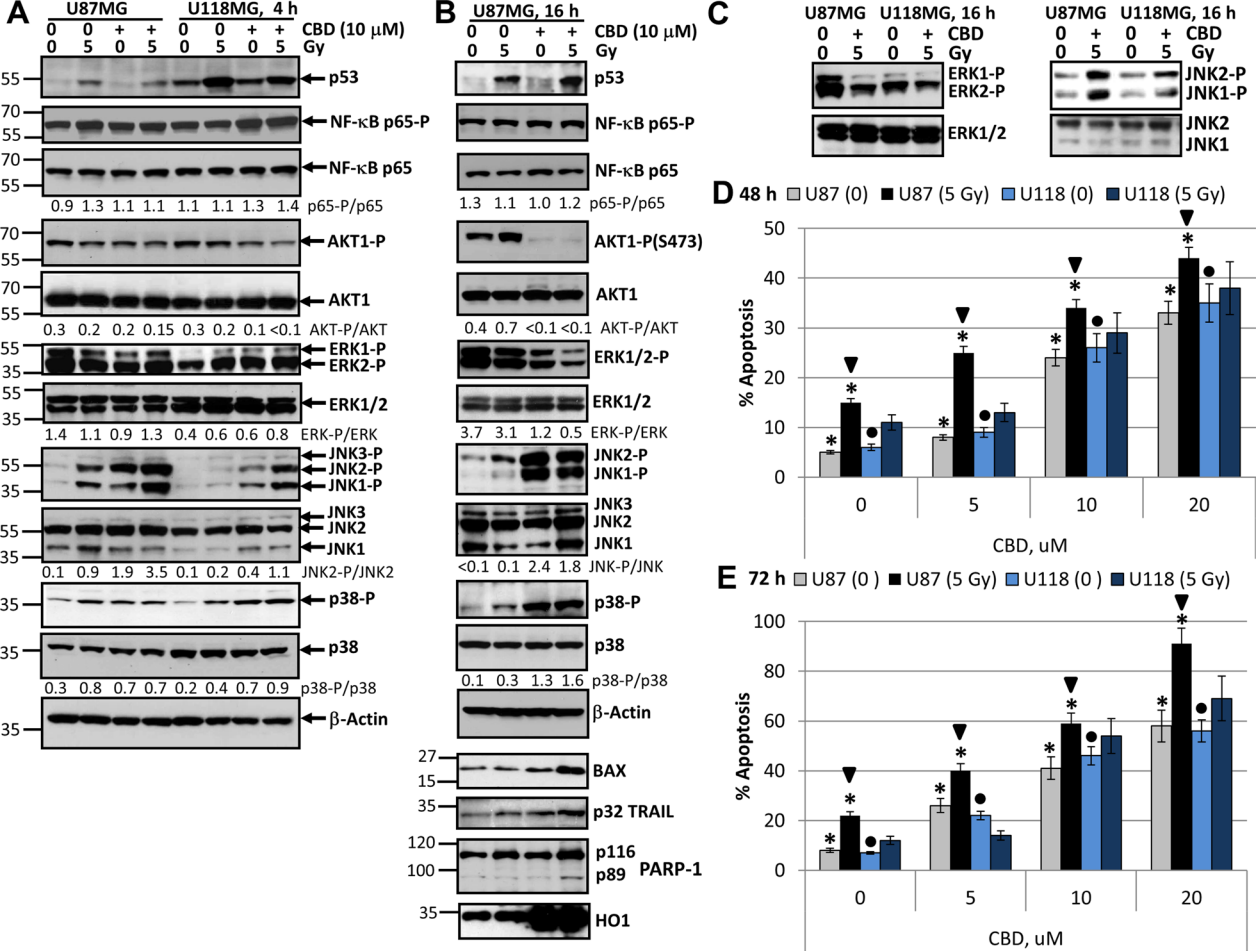
Changes in cell signaling proteins and induction of apoptosis in U87MG and U118MG glioblastoma cells following treatment with γ-irradiation and CBD, alone or in combination (**A**, **B**, **C**) Western blot analysis of cell signaling proteins 4 h and 16 h after combined treatment of U87MG and U118MG cells with CBD (10 µM) and γ-irradiation (5 Gy). CBD was diluted in DMSO; 0.1% DMSO served as a vehicle; CBD and DMSO were added to the cell cultures 30 min before irradiation. (**D**, **E**) Cell cycle-apoptosis analysis of U87MG and U118MG cells 48 and 72 h after combined treatment with increased concentrations of CBD and γ-radiation (5 Gy). CBD (diluted in DMSO) was added 30 min before irradiation. Control cells (CBD, 0) were treated with 0.1% DMSO 48–72 h after treatment; then cell nuclei were stained with PI and DNA content was determined using the flow cytometry. Apoptotic cells and secondary necrotic cells (originated from apoptotic cells) were in the pre-G1 region. Pooled results of four independent experiments of dose-dependent effects of CBD and irradiation (5 Gy) for U87MG and U118MG cells are shown. Error bars represent means ± S.D. (*p* < 0.05, Student’s *t*-test). Stars indicate significant differences in apoptotic levels between non-irradiated and irradiated U87MG cells; arrows indicated significant differences between irradiated U87MG cells pending CBD concentration; circles indicate significant differences in apoptosis between non-irradiated U118MG cells in the presence of CBD.

Since U87MG glioblastoma cells contained a normal variant of p53 while U118MG had mutated p53, we expected a different response of these cell lines to combined treatment, due to decreased activity of the mitochondrial apoptotic pathway in U118MG cells. First, we determined changes in the levels and activities of the main signaling proteins at 4 h and 16 h after treatment with 10 µM CBD and γ-radiation at 5 Gy, alone or in combination (Figure 1A–1C), and then the late events (48–72 h) linked with cell death by apoptosis in U87MG and U118MG cells after dose-dependent-treatment by CBD (5–20 µM) and γ-irradiation (5 Gy) (Figure 1D, 1E). CBD in 0.1% DMSO or control 0.1% DMSO were added to the cell cultures 30 min before irradiation. Change of p53 protein levels demonstrated efficacy of γ-irradiation: in both GBM lines, U87MG with the normal p53 and U118MG with a mutated and more stable variant of p53, protein levels of p53 were additionally upregulated after irradiation (Figure 1A, 1B). T98G cells also had mutated p53 and destroyed mitochondrial apoptotic pathway [46].

The active phospho-Ser568-p65 NF-κB levels were relatively stable in U87MG and slightly increased after CBD or combined treatment (4 h) of U118MG cells (Figure 1A). A modest downregulation of the high basal AKT1 activity (represented by phospho-Ser473-AKT1 levels) was detectable 4 h after treatment with CBD, alone or in combination with radiation, in both lines. Phospho-ERK1/2 levels were also relatively stable in U87MG cells 4 h after indicated treatments while these levels demonstrated modest increase after CBD and γ-irradiation treatments in U118MG cells (Figure 1A). In contrast, upregulation of phospho-JNK1/2 levels was observed in both GBM lines 4 h after treatment with γ-irradiation or CBD, alone or, especially, in combination, but with the characteristic difference between these lines: a dramatic increase of phospho-JNK1/2 levels in U87MG cells and less pronounced and variable increase in U118MG cells (Figure 1A). Phospho-(T180/Y182)-p38 MAPK levels modestly increased 4 h after treatment with CBD or γ-irradiation in both cell lines (Figure 1A).

Signaling events in U87MG cells after 16 h exposure with CBD and γ-radiation included upregulation of p53 but relatively stable levels of total and active NF-κΒ phospho-Ser536-p65 (Figure 1B). Substantial changes observed after 16 h of treatment also included a strong downregulation of phospho-AKT levels (Figure 1B), a notable downregulation of phospho-ERK1/2, a significant upregulation of phospho-JNK1/2 and phospho-MAPK p38 levels. Pronounced negative effects of CBD treatment on AKT and ERK1/2 activities were previously described [26, 47], however, we would like to highlight a strong activation of JNK1/2 and MAPK p38 in these conditions (Figure 1B). On the other hand, a modest upregulation of JNK1/2 and p38 activities and a substantial increase in p53 protein levels were characteristic for U87MG cells after γ-irradiation alone. In general, synergistic effects of CBD and γ-irradiation on the main signaling proteins were observed for the early upregulation of active JNK1/2, the late downregulation of ERK1/2 activities and the late upregulation of proapoptotic proteins, BAX and TRAIL (Figure 1B). The significance of TRAIL expression for cell death regulation will be elucidated in the subsequent experiments. The late activation of MAPK p38 was dependent most from CBD while impact of γ-irradiation was modest. Additionally, caspase-3-dependent cleavage of PARP-1, an indicator of apoptotic commitment, was well pronounced 16 h after combined treatment of GBM cells (Figure 1B). A substantial upregulation of heme oxygenase-1 (HO1) levels that was indicative of protection activity against oxidative stress was observed 4–16 h after treatment of U87MG cells by CBD alone or in combination with radiation (Figure 1B). A difference in the levels of inducible JNK1/2 activities between U87MG and U118MG cells was maintained after overnight treatment; furthermore, levels of ERK1/2 activities were decreased in both glioma lines after overnight treatment (Figure 1C).

Dose-dependent effects of CBD (5–10 µM) on induction of apoptosis occurred with relatively similar kinetics in both GBM lines (Figure 1D, 1E). However, additional significant effects of γ-irradiation (5 Gy) on CBD-induced apoptosis 48 h and 72 h after treatment were detected only in U87MG cells, probably, due to the intact p53-BAX pro-apoptotic system in these cells. After 48 h of combined treatment (20 µM CBD and 5 Gy) apoptotic levels were close to 50%, and after 72 h they achieved almost 90% in U87MG cells and almost 70% in U118MG cells (Figure 1D, 1E). In general, CBD-induced cell death by apoptosis was a slowly developing process in gliomas suggesting the participation of the secondary mechanism of amplification of cell death.

**Figure 2:**
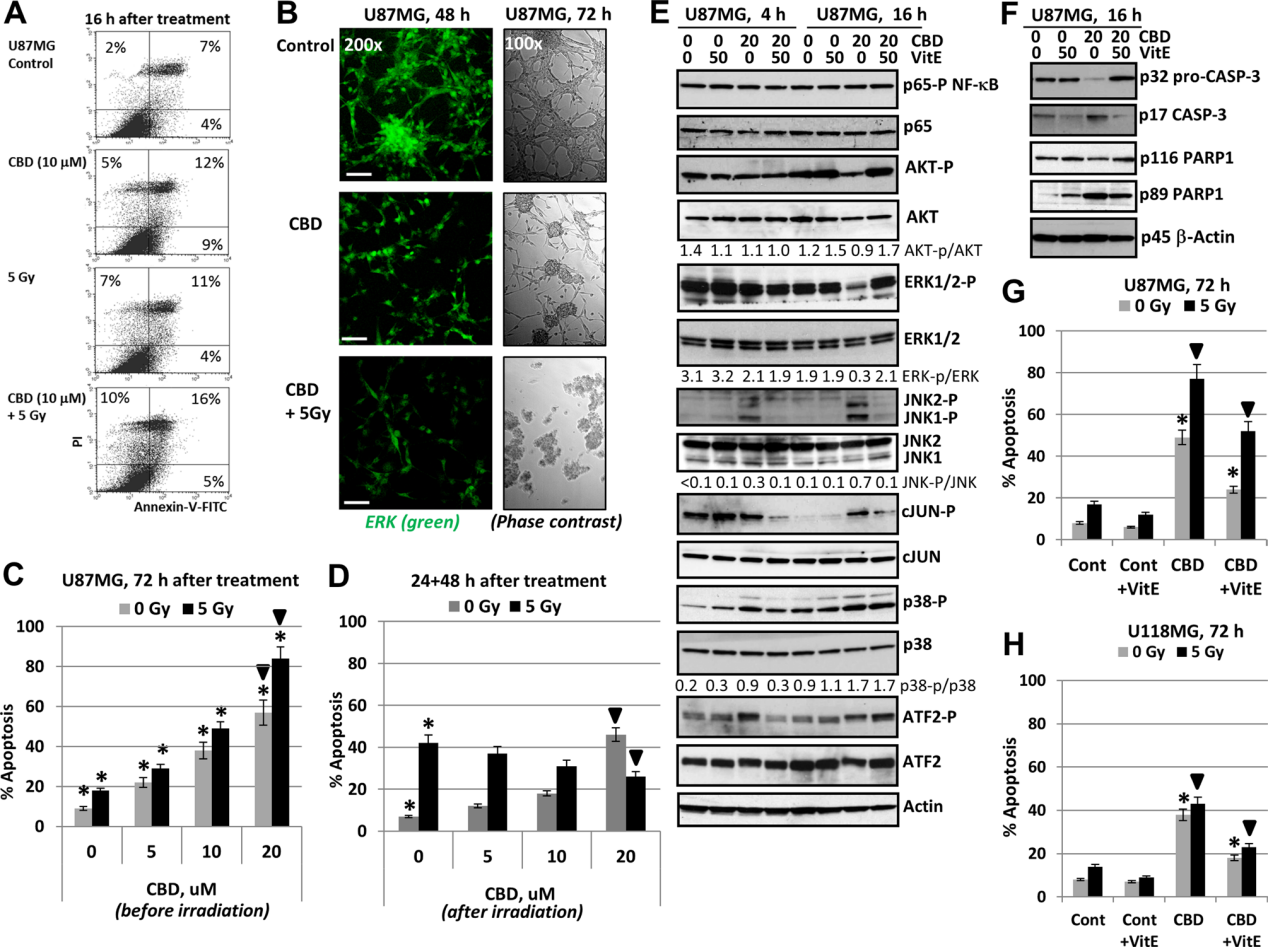
Regulation of cannabidiol (CBD)- induced apoptosis in human U87MG glioblastoma cells (**A**) Annexin-V-FITC and PI staining of U87MG glioblastoma cells 16 h after treatment by CBD and γ-irradiation (5 Gy) alone or in combination was performed with the subsequent FACS analysis. (**B**) Immunostaining of total ERK (48 h) and phase-contrast microscopy of U87MG cells 48 h (72 h) after indicated treatment. Typical images are shown. Bar = 50 μm. (**C**) Dose-dependent effect of co-treatment by CBD on radiation-induced apoptosis (5 Gy) in U87MG glioblastoma cells. CBD in DMSO solution was added 30 min before irradiation. Cell cycle-apoptosis analysis was performed using PI staining of DNA and flow cytometry. Pooled results of four independent experiments are shown 72 h after treatment. Error bars represent means ± S.D. *(p* < 0.05, Student’s *t*-test). Stars indicate a significant difference between non-irradiated and irradiated cells; arrows indicate a significant effect after combined treatment with CBD (20 µM) and γ-irradiation (5 Gy). (**D**) U87MG cells were first irradiated at 5 Gy; 16 h after irradiation CBD and DMSO were added for an additional 48 h. Stars indicate a significant difference between non-irradiated and irradiated cells; black arrows indicate a significant negative effect after combined treatment with CBD (20 µM) and γ-irradiation (5 Gy). (**E**, **F**) Effect of antioxidant Vitamin E (50 µM) on CBD-induced signaling in U87MG cells 4,16 and 48 h after CBD treatments; Vitamin E (VitE) in ethanol was added 15 min before CBD. Western blot analysis was performed using the standard procedures. (**G**, **H**) Effects of VitE (50 µM) on apoptosis induced by CBD or combined treatment of CBD and γ-irradiation in U87MG and U118MG cells. Stars indicate a significant down-regulation of CBD-induced apoptosis in the presence of Vitamin E (50 µM); arrows indicate a significant down-regulation of apoptosis after combined treatment with CBD (20 µM) and γ-irradiation (5 Gy) in the presence of VitE.

However, it was possible to detect the early effects of CBD and γ-irradiation in the induction of apoptosis for U87MG cells 16 h after exposure. Indeed, beside caspase-3-dependent PARP-1 cleavage (Figure 1B), Annexin-V-FITC and PI staining followed by FACS analysis demonstrated low but statistically significant apoptotic and the secondary necrotic levels (Annexin-V-positive and Annexin-V-negative, respectively) 16 h after CBD treatment (at 10 µM) or γ-irradiation (5 Gy) alone or, especially, in combination (Figure 2A). Immunostaining of adherent glioma cells using anti-total ERK Ab (the left panel of Figure 2B) and phase-contrast microscopy of the native glioma cell cultures (the right panel) did not reveal notable changes in cell morphology 16 h after treatment but demonstrated strong death-induced effects of CBD 48–72 h after treatment including damage of cell-cell interaction and appearance of dead cell bodies (not neurospheres). Finally, combined treatment (after 48–72 h) resulted in low cell survival and strong death-related changes in the cell cultures (Figure 2B).

Interestingly, there was a critical difference in the consequence of combined treatment of GBM cells depending on the sequence of treatment. So, we observed strong effects of CBD pretreatment that occurred 30 min before exposure to γ-radiation on the final levels of apoptosis (Figure 2C). A change of the order of treatment of U87MG cells using the initial γ-irradiation (10 Gy) and delayed addition of CBD (24 h after irradiation) lead to an actual downregulation of radiation-induced apoptosis by CBD (Figure 2D). The significance of this phenomenon will be investigated in additional experiments.

As was previously demonstrated using glioma cells, the primary action of CBD included a substantial upregulation of ROS production [40, 48]. Using lipid-soluble antioxidant α-Tocopherol (Vitamin E; VitE) that could penetrate into the glioma cells through the cell membrane at a dose 50 µM, we observed substantial suppression of CBD-induced apoptosis in U87MG and less pronounced in U118MG cells, as was previously reported [40, 48], and partial suppression of apoptosis induced by combined treatment of CBD and γ-irradiation in both cell lines (Figure 2G, 2H). At the levels of signaling proteins, we observed a stable NF-κB p65-P activity together with restoration of CBD-induced downregulation of phospho-AKT and phospho-ERK levels 16 h after co-treatment of CBD and VitE (Figure 2E). Interestingly, VitE strongly downregulated CBD-induced JNK1/2-cJUN phosphorylation 4–16 h after co-treatment while its effect on downregulation of MAPK-p38-ATF2 phosphorylation was modest and only 4 h after treatment (Figure 2E). Results obtained further indicated a distinctive role of JNK, as well as MAPK p38, ERK and AKT in mediation of CBD-induced oxidative stress and may serve as a foundation for the subsequent experiments. Furthermore, VitE co-treatment suppressed activation of caspase-3 via decreased p17 (active form) production and partially downregulated caspase-3-mediated PARP1 cleavage (Figure 2F). Our data demonstrated partial protective effects of VitE against CBD- and (CBD+5Gy)-induced apoptosis in U87MG cells (Figure 2G) as well as in U118MG cells (Figure 2H).

**Figure 3:**
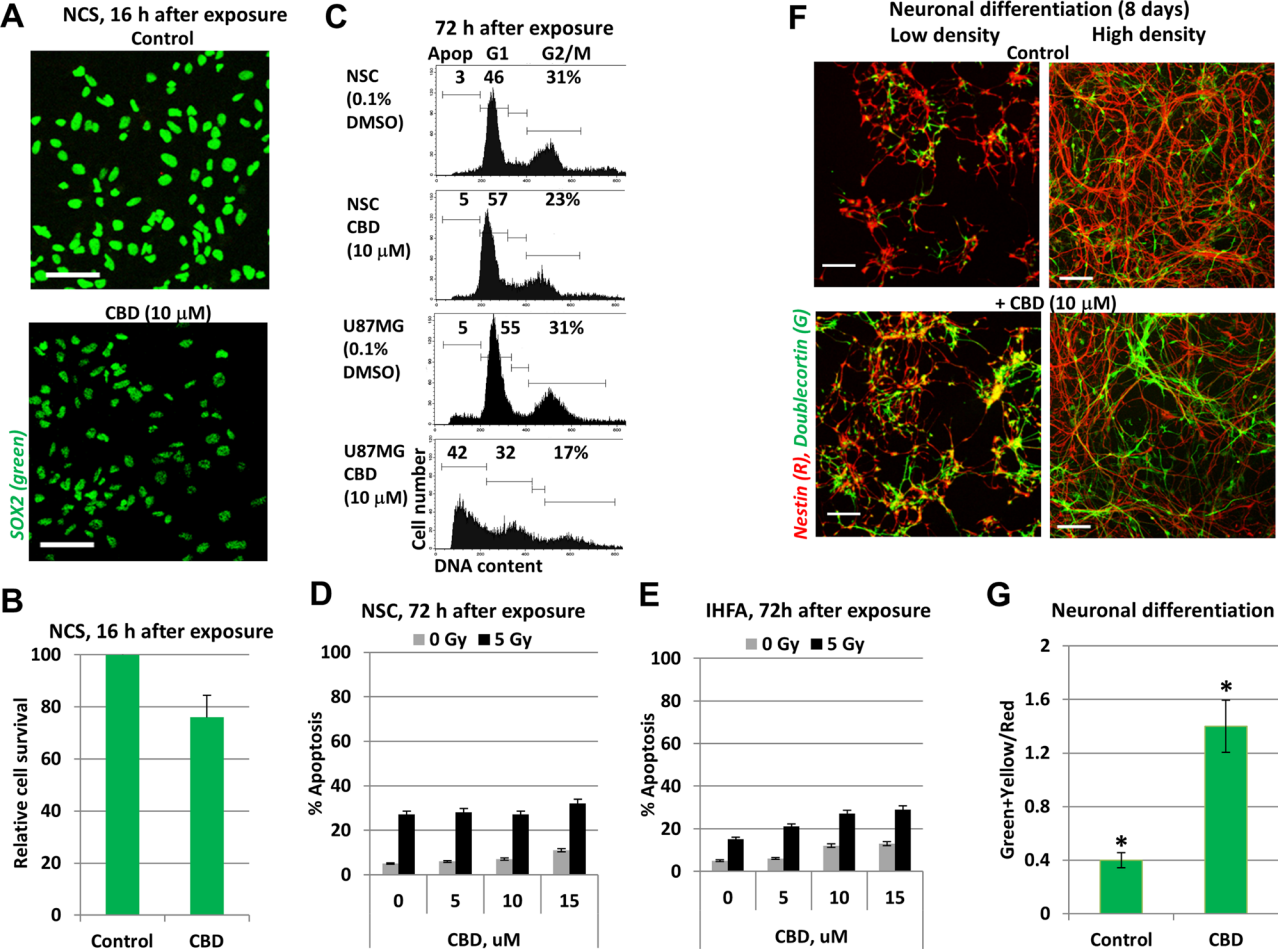
CBD treatment did not induce death of neural stem/progenitor cells (NSC/NPC) and astrocytes (**A**, **B**) Relative survival of NSC/NPC was determined 16 h after CBD (15 µM) treatment. NSC/NPC was immunostained using anti-SOX2 Ab. (**C**) The typical result of cell cycle-apoptosis analysis demonstrates differential effects of CBD (10 µM) on upregulation of apoptosis in U87MG cells and NSC/NPC 72 h after treatment. CBD was dissolved in DMSO; 0.1% DMSO was added to the control cultures as a vehicle. Cell cycle-apoptosis analysis was performed using PI staining of DNA and flow cytometry. (**D**) Radiation-induced (5 Gy) apoptotic levels in NSC were not changed after co-treatment with increasing concentration of CBD. Apoptotic levels were determined using PI staining of DNA and sub-G1 analysis. (**E**) Apoptotic levels after combined treatment of immortalized human embryonic astrocytes (IHEA). (**F**, **G**) Positive effects of CBD (10 µM) on neuronal differentiation of NSC/NPC in cell culture conditions. Induction of differentiation was performed for NCS/NPC at low or high density in the presence or absence of CBD. Confocal analysis of immunofluorescent images was done using monoclonal Ab against Nestin, an early neuroprogenitor marker (red), and polyclonal Ab against Doublecortin, a neuronal marker (green). Bar = 50 μm. Green+yellow/Red cells ratio indicate a degree of neuronal differentiation. Stars indicate a significant difference between control and CBD-treated cells.

In contrast to glioblastoma cells, human neural stem/progenitor cells (NSC/NPC), which were also known as the ancestors of glioma/glioblastoma (Figure 3A–3C), and immortalized human fetal astrocytes (IHFA) did not respond by induction of notable levels of apoptosis following CBD treatment. Furthermore, ionizing irradiation at 5 Gy in combination with 5–15 µM CBD (added 0.5 h before irradiation), which notably increased radiation-induced apoptotic death levels in U87MG cells (see Figure 1D and 1E), did not significantly change the initial radiation-induced death levels for NSC/NPC and caused only modest alteration in apoptotic levels of immortalized human fetal astrocytes (IHFA) (Figure 3D, 3E). Interestingly, CBD treatment (10 µM) caused downregulation of SOX2 levels in NSC/NPS (Figure 3A). It was followed by acceleration of neuronal differentiation of NSC/NPC (treated at low or high cell density), as was determined by an increased ratio of Doublecortin (green), a neuronal marker, to Nestin (red), an early neuroprogenitor marker (Figure 3F, 3G). The similar effects on neuronal differentiation were previously observed for endocannabinoids [34]. Hence, there was an obvious preference of CBD to use different programs for initiation of apoptotic cell death in GBM cells and for control of the normal functions in NSC/NPC and astrocytes. This observation could be a basis for experiments *in vivo* to assess the optimal ratio between CBD and a dose of γ-irradiation for combined treatment of glioblastoma with minimal damaging effects for normal cells in the brain.

**Figure 4:**
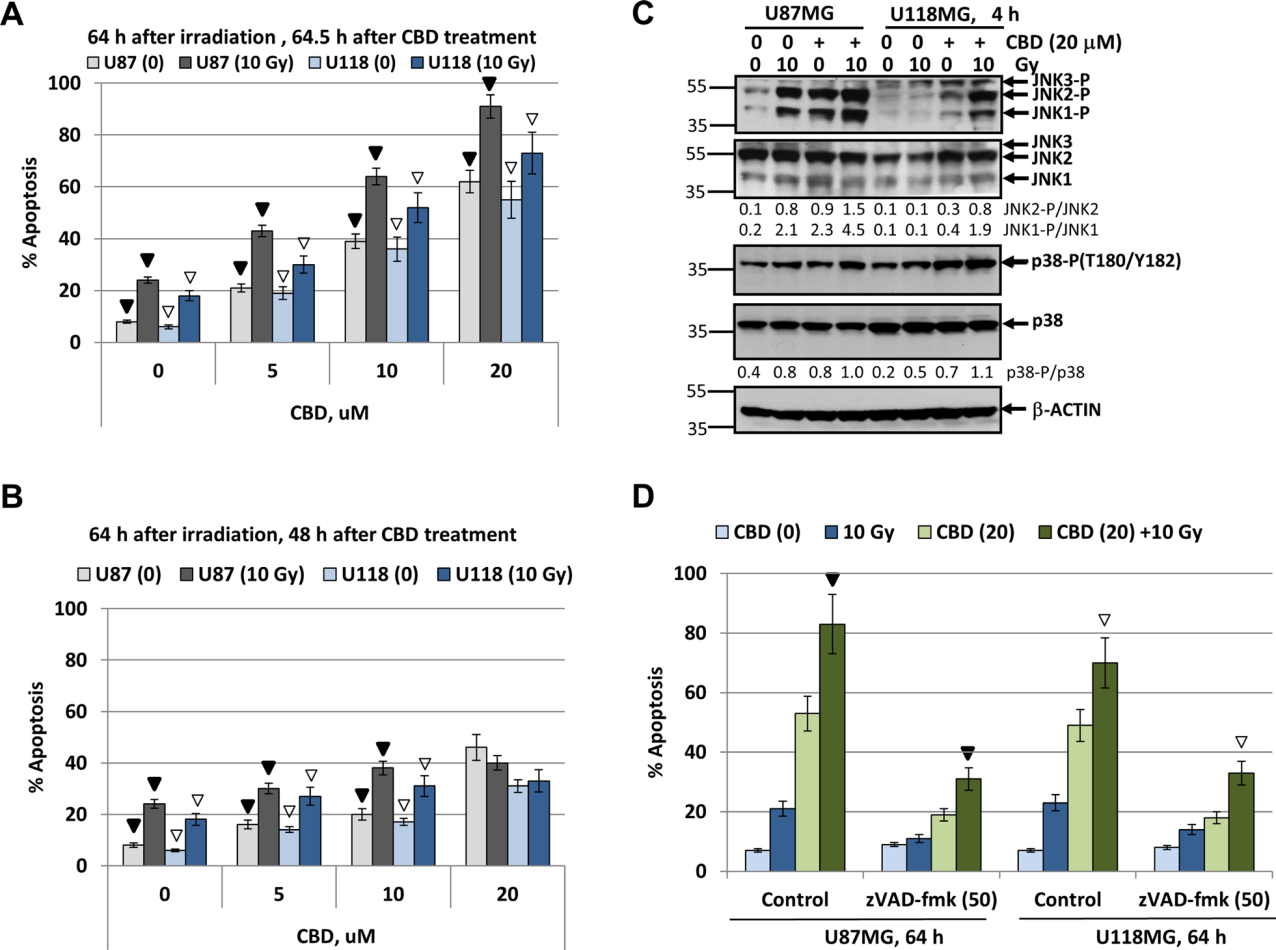
Upregulation of radiation-induced apoptosis in U87MG and U118MG glioblastoma cells by CBD is dependent from CBD pretreatment before irradiation (**A**, **B**) Cell cycle-apoptosis analysis of U87MG and U118MG cells 64 h after indicated treatments. The experiments were performed as described in Figure 2C and 2D but with an increased final concentration of CBD (20 µM) and dose of γ-irradiation (10 Gy). CBD was added to the cell cultures 30 min before irradiation for panel A or 24 h after irradiation (for panel B). Cells were stained with PI, and DNA content in the cell nuclei was determined using the flow cytometry. Effects of small molecule inhibitors, SP600125 (20 µM) and SB203580 (20 µM), on CBD-induced (20 µM) apoptosis was detected. The pooled results of four independent experiments are presented. Error bars represent means ± S.D. *(p* < 0.05, Student’s *t*-test). In panel A, black arrows indicate a significant increase in the apoptotic levels of U87MG cells pre-treated by 0–20 µM CBD that followed by γ-irradiation (10 Gy); open arrows indicate similar changes in U118MG cells. In panel B, black arrows and open arrows indicate significant differences in radiation-induced apoptotic levels after treatment by CBD 24 h after irradiation for U87MG and U118MG, respectively. (**C**) Western blot analysis of signaling proteins after treatment of U87MG and U118MG cells by CBD (20 µM) and γ-irradiation (10 Gy), alone or in combination. CBD pretreatment was performed 0.5 h before γ-irradiation. (**D**) Effect of pancaspase inhibitor zVAD-fmk (50 µM) on apoptosis induced by combined treatment of CBD (20 µM) and γ-irradiation (10 Gy). Black arrow indicates significant effects of zVAD-fmk on apoptosis in U87MG cells, open arrows - in U118MG cells.

Since high total doses of γ-irradiation (46–60 Gy) were used in clinics for radiotherapy of human glioblastoma [49], we further assessed killing effects of CBD and γ-irradiation on glioblastoma cells following treatment at increased radiation dose (10 Gy) and CBD (20 µM). In these conditions, we observed an additional upregulation of CBD-induced apoptosis not only in U87MG cells but also in U118MG cells if CBD pretreatment was before irradiation (Figure 4A). On the other hand, delayed addition of CBD 16 h after irradiation again down-regulated radiation-induced apoptosis in both glioblastoma lines (Figure 4B). Western blot analysis further confirmed well-pronounced upregulation of JNK and moderate increase of MAPK p38 activities in glioblastoma lines after such treatments (Figure 4C).

We used a pancaspase inhibitor zVAD-fmk (50 µM in 0.1% DMSO) to suppress apoptotic cell death induced CBD (20 µM) alone or in combination with irradiation (10 Gy) (Figure 4D). Pretreatment with zVAD-fmk, which was added to the glioblastoma medium 1 h before treatment, substantially downregulated % of pre-G1 cells with apoptotic nuclei, further confirming apoptotic commitment after combined treatment of glioblastoma cells under high stress conditions. Levels of total cell death resistant to zVAD-fmk treatment might include secondary necrotic cells. However, co-treatment with Necrostatin (50 µM), an inhibitor of RIP1 kinase and necroptosis [50], did not notably change total cell death levels in CBD-treated glioma cells (data not shown), highlighting the absence of the major role for programmed necrosis/necroptosis in these conditions.

### Differential role of ERK, MAPK p38, JNK and AKT in modulating CBD-induced death of glioblastoma cells

**Figure 5:**
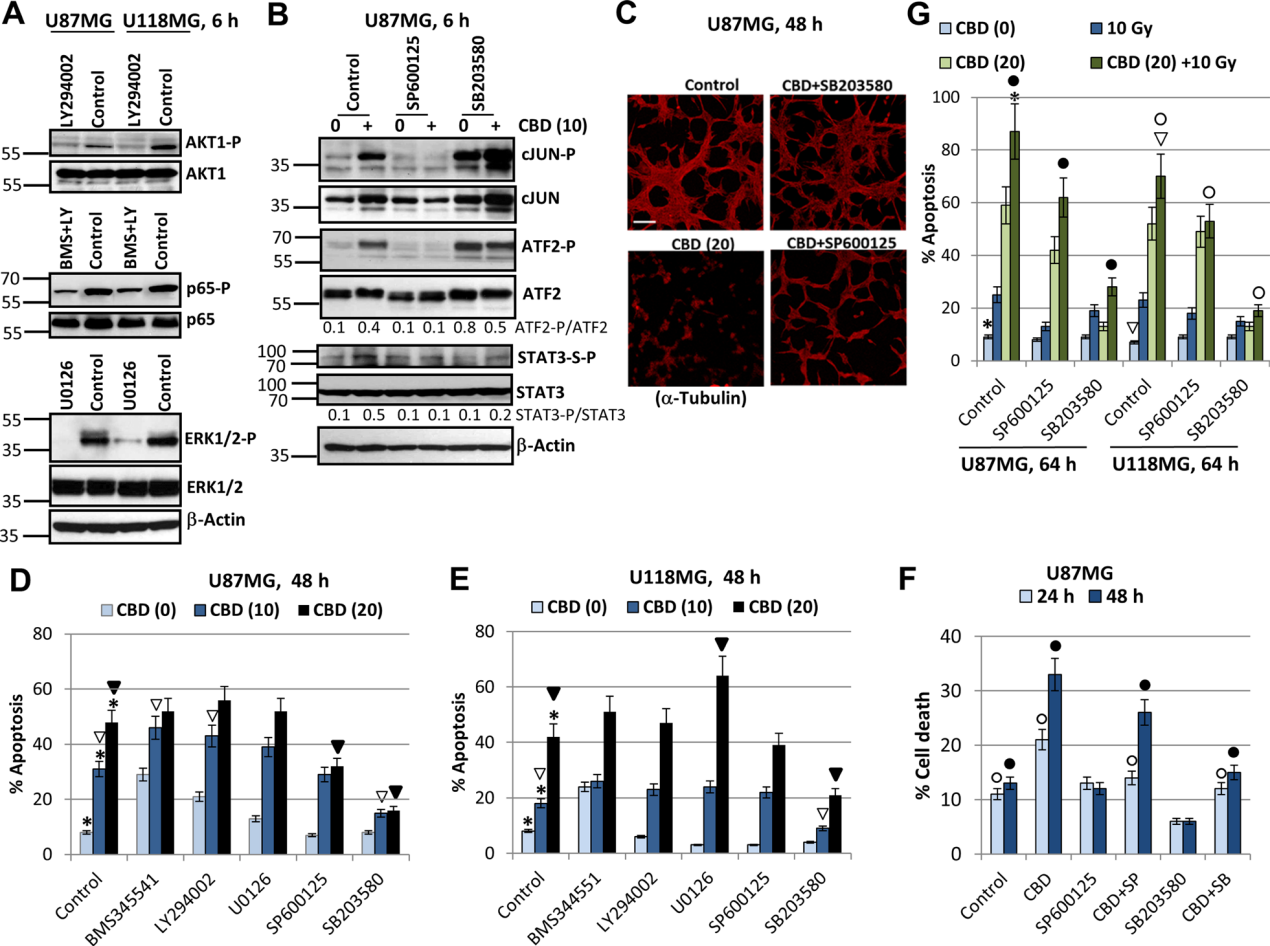
Effects of small molecule inhibitors of cell signaling pathways on modulation of CBD-induced apoptosis in U87MG and U118MG glioblastoma cells (**A**) Effects of small molecule inhibitor of PI3K, LY294002 (50 µM); IKKβ, BMS345541 (20 µM); MEK1/2, U0126 (10 µM); and JNK1-3, SP600125 (20 µM) on the corresponding downstream targets were determined using Western blot analysis. (**B**) Effect of small molecule inhibitors of JNK, SP600125 (20 µM), and MAPK p38, SB303580 (20 µM) on their targets in U87MG cells. (**C**) Effects of small molecule inhibitors on CBD-induced cell death. Immunostaining of U87MG cells (48 h after treatment) with anti-α-Tubulin mAb was performed. Bar = 50 µm. (**D**, **E**) Cell cycle-apoptosis analysis of U87MG and U118MG cells 48 h after indicated treatments. Cells were stained with PI, and DNA content in the cell nuclei was determined using the flow cytometry. Effects of specific molecule inhibitors, BMS345541 (20 µM), LY294002 (50 µM), U0126 (10 µM), SP600125 (20 µM) and SB203580 (20 µM), on CBD-induced (10–20 µM) apoptosis were detected. The pooled results of four independent experiments are presented. Error bars represent means ± S.D. *(p* < 0.05, Student’s *t*-test). Stars indicate a significant difference between control (0.2% DMSO) and CBD-treated glioblastoma cells; open arrows - between CBD (10 µM) and (CBD + inhibitor) treated cells; black arrows indicate a significant decrease in the apoptotic levels between CBD (20 µM)- and (CBD + inhibitor)-treated cells. (**F**) Effects SP600125 (20 µM) and SB203580 (20 µM) on CBD-induced (10 µM) cell death were determined. The pooled results of four independent experiments are presented. Error bars represent means ± S.D. *(p* < 0.05, Student’s *t*-test). Open and black circles indicate a significant difference between control (0.2% DMSO), CBD-treated and (CBD + inhibitor)-treated cells after 24 h and 48 h, respectively. (**G**) Effect of small molecule inhibitors of JNK, SP600125 (20 µM), and MAPK p38, SB303580 (20 µM) on apoptosis induced by combined treatment of CBD and γ-irradiation (10 Gy). The pooled results of four independent experiments are presented. Error bars represent means ± S.D. *(p* < 0.05, Student’s *t*-test). Stars and arrows indicate a significant increase in apoptotic levels induced by combined treatment in U87MG and U118MG cells, respectively. Black circles indicate significant effects of small molecule inhibitors on apoptosis after combined treatment in U87MG cells, open circles - in U118MG cells.

What is the role of cell signaling mechanisms, which are involved in modulating cell survival and apoptosis in GBM after CBD treatment? To address this question, we assessed effects of small molecule inhibitors of several crucial signaling pathways on CBD-induced signaling cascades: 1) BMS34554, an inhibitor of IKKβ, that protected IκΒ from degradation resulting in down-regulation of NF-κB nuclear translocation, a decrease of the active nuclear NF-κB p65 levels and suppression of p65-Ser536 phosphorylation; 2) LY294002, an inhibitor of PI3K, that suppressed the downstream AKT1 phosphorylation/ activation and finally affected numerous AKT downstream targets, including mTORC1 and IKKα; 3) U0126, an inhibitor of MEK1/2, that suppressed downstream ERK1/2 phosphorylation and activation; 4) SP600125, an inhibitor of the enzymatic activity of JNK1-3 with the downstream suppression of cJUN phosphorylation; and 5) SB203580, an enzymatic inhibitor of MAPK p38 activity that could suppress activities of numerous downstream targets. Based on the results of our previous publication [22], we observed specific inhibition of AKT1 phosphorylation by LY294002 (50 µM); a downregulation of NF-κB p65-Ser536 phosphorylation only by combined treatment of BMS344551 (20 µM) and LY294002 (50 µM); specific inhibition of ERK1/2 phosphorylation by U0126 (10 µM) in both glioblastoma lines (Figure 5A).

Next, we were focused on JNK and MAPK p38 signaling pathways and their downstream targets, such as transcription factors cJUN and ATF2. Strong upregulation of JNK-P and relatively moderated increase of MAPK p38-P activities were characteristic features of CBD treatment in U87MG cells accompanied by pronounced increase of phospho-JUN and phospho-ATF2 activities (see Figure 1A, 1B and Figure 5B). SP600125 (20 µM, an inhibitor of JNK1-3 enzymatic activity specifically suppressed phosphorylation of cJUN-P(S73), primary target of JNK, as well as ATF2-P(T71), a secondary target for JNK. Surprisingly, the vast majority of ATF2-P(T71) phosphorylation was controlled by JNK rather than MAPK p38 in U87MG cells and was strongly downregulated in the presence of SP600125 (Figure 5B). A complicated and quite confusing consequence of usage of SB203580 (20 µM), a specific MAPK p38α/p38β enzymatic inhibitor, was a rapid and strong upregulation of the compensatory JNK activity and the basal levels of phospho-cJUN and phospho-ATF2 by JNK-mediated phosphorylation [51]; see Figure 5B. The presence of SB203580 only modestly decreased CBD-induced levels of STAT3-Ser727-P (an additional target of MAPK p38), compared to control cells (Figure 5B).

On the other hand, we observed that SB203580 (20 µM) protected U87MG cells from CBD-induced cell death and efficiently maintained cell-cell interactions and the traditional 2D architecture in U87MG culture while SP600125 (20 µM) demonstrated only partial protection (Figure 5C). We further demonstrated that SB203580 (20 µM) effectively down-regulated apoptosis induced by CBD (10–20 µM) in both U87MG and U118MG cells (48 h after treatment), while SP600125 (20 µM) showed only moderate negative effects on CBD-induced apoptosis in U87MG cells (Figure 5D, 5E), highlighting a probable leading role of MAPK p38-P for regulation of CBD-induced apoptosis in both glioma cell lines. Interestingly, determination of total levels of cell death (by Trypan blue staining and light microscopy) showed modest protective effect of JNK1/2 inhibition 24–48 h after CBD treatment while protective effects of MAPK p38 inhibition were well-pronounced at these time points (Figure 5F).

We observed, furthermore, statistically significant effects of BMS345541 and LY294002 on upregulation of CBD-induced apoptosis (at CBD dose 10 µM but not 20 µM) in U87MG cells (Figure 5D). U0126 upregulated CBD-induced apoptosis in U118MG cells (at the CBD dose 20 µM), while additional effects of BMS345541 or LY294002 alone on CBD-induced apoptosis in U118MG cells were only marginal (Figure 5E). A positive role of the EGFR-MEK-ERK pathway in the survival of GBM cells and the negative regulation of this pathway by CBD was demonstrated in previous publications [26, 47] and were further confirmed in the present study by demonstration of enhancing effects of U0126 on CBD-induced apoptosis in U118MG cells.

We further assessed effects of a small molecule inhibitor of JNK1-3, SP600125 (20 µM), and inhibitor of MAPK p38, SB203580 (20 µM), on apoptosis in U87MG and U118MG cells after treatment at increased stress conditions with CBD (20 µM) and γ-irradiation (10 Gy) (Figure 5G). In U87MG and U118MG cells, SB203580 strongly suppressed the effects of CBD, alone or in combination with irradiation, on upregulation of apoptotic levels. On the other hand, suppressive effects of SP600128 on apoptosis after combined treatment in both cell lines were significant, even less pronounced than effects of SB203580, emphasizing again an important role of MAPK p38 for induction of apoptosis after CBD treatment (alone or in combination with γ-radiation) and a supplementary role for JNK in the enhancement of apoptosis. A cross-talk between stress kinases, MAPK p38 and JNK, appears to be critical for apoptotic regulation in glioblastoma cells. Actually, high level of activation of JNK by CBD could initiate JNK-mediated autophagy in glioma cells [52]. Cross-talk of autophagy and apoptosis could result either in suppression or in upregulation of apoptosis in glioma cells, due to unknown regulatory mechanism.

**Figure 6:**
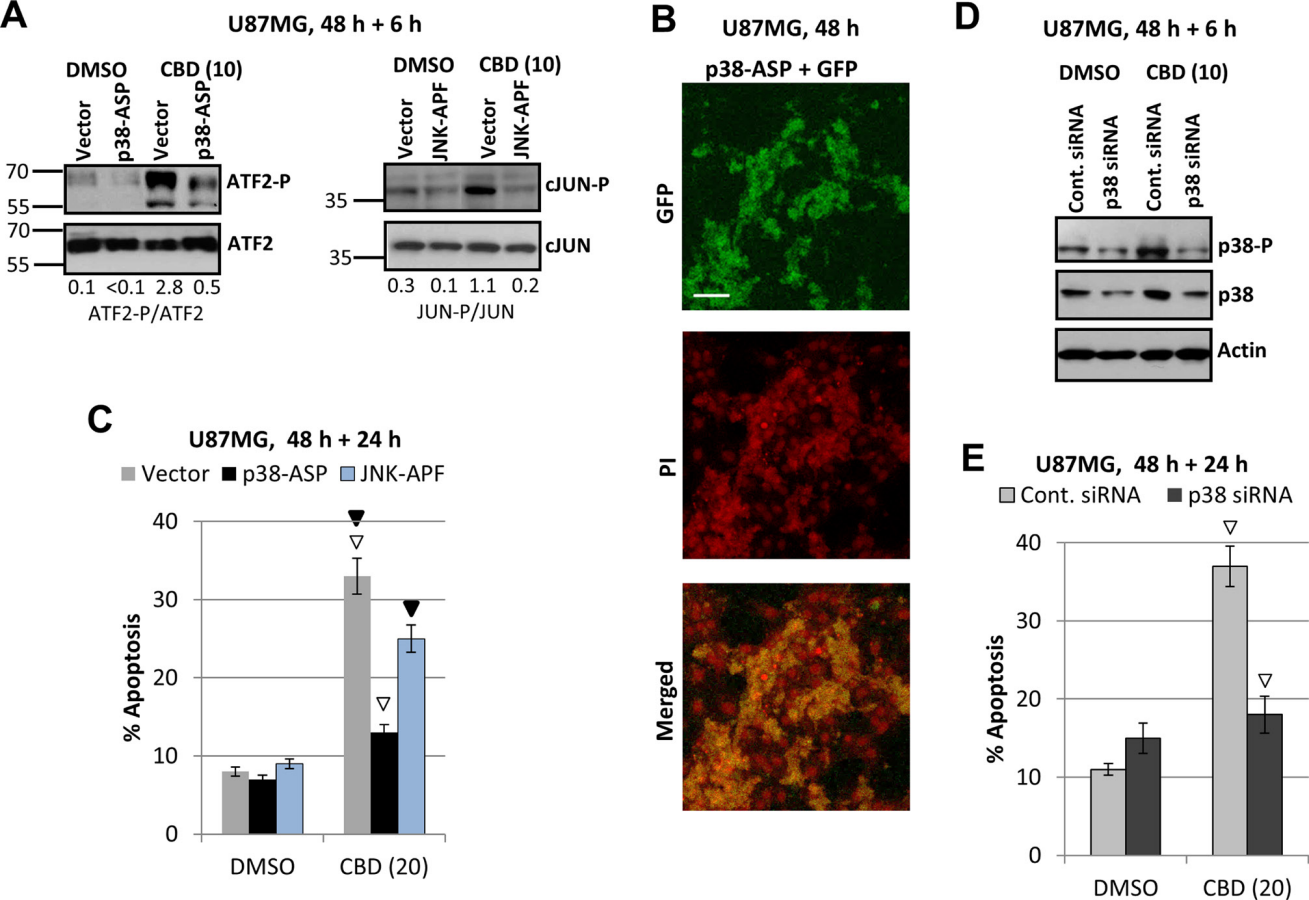
Downregulation of ATF-P and cJUN-P levels and silencing MAPK p38 expression: effects on CBD-induced apoptosis **(A)** Effects of CBD (10 µM; 6 h) on ATF2-P levels in U87MG cells transiently transfected with p38-ASP construct + GFP; effects of CBD on cJUN-P levels after transient transfection with dominant-negative JNK-APF + GFP. (**B**) Efficiency of transfection was determined using confocal microscopy for detection GFP in PI-stained nuclei of U87MG cells. (**C**) In the parallel experiments, the apoptotic levels among green U87MG cells transiently transfected by the empty vector + GFP, p38-ASP + GFP or JNK-APF + GFP were determined 48 h after transfection and 24 h after DMSO or CBD treatment using the flow cytometry. Open arrows indicate a significant decrease in CBD-induced apoptosis for p38-ASP transfected cells, black arrows for JNK-ARF-transfected cells. (**D**) Downregulation of MAPK p38 expression using p38 siRNA. CBD treatment (additional 6 h) was performed 48 h after transfection. (**E**) In the parallel experiments, the apoptotic levels among green U87MG cells transiently transfected by control siRNA+Green Fluorescein Conjugate and p38 siRNA+Green Fluorescein Conjugate were determined 48 h after transfection and 24 h after DMSO or CBD treatment using the flow cytometry. Open arrows indicate a significant decrease in CBD-induced apoptosis for p38 siRNA transfected cells.

Due to uncertainty of a role for ATF2 as the primer MAPK p38-P target in U87MG, we used alternative approaches to further elucidate a role of MAPK p38 in the regulation of CBD-induced apoptosis: (i) a transient transfection of dominant negative p38-ASP construct (in the presence of GFP expression vector) that notably downregulated active ATF2-P levels in CBD-treated cells (Figure 6A); (ii) a transient transfection of dominant-negative JNK1-ARF + GFP that notably downregulated active CBD-induced cJUN-P levels (Figure 6A). An efficiency of transfection of U87MG cells (around 60–70%) is demonstrated on Figure 6B. In parallel experiments, we determined the levels of apoptosis (by PI staining of apoptotic nuclei and detection of % pre-G1 cells) among green (GFP-positive) transfected cells using flow cytometry. Results obtained in these experiments demonstrated a significant downregulation of apoptotic levels in transfected cells with deficiency of p38-P 48 h after indicated treatments, emphasizing a role of active MAPK p38 in the regulation of cell death (Figure 6B). A dominant negative p38-ASP construct (Figure 6A, 6B) was previously used in our study of melanoma apoptosis [53, 54]. Dominant-negative construct JNK1-APF partially suppressed cJUN phosphorylation in transfected cells after CBD treatment (Figure 6A) and demonstrated a modest downregulation of apoptosis after combined treatment of U87MG cells (Figure 6B).

Additionally, we performed a partial silencing total p38 levels in U87MG cells using transfection of the control and p38 siRNA in the presence of Fluorescein Conjugate (at a ratio 5:1) (“Cell Signaling”), see Figure 6D, 6E. A down regulation of total p38 and the corresponding p38-P levels (6 h after CBD treatment) was accompanied by decreased levels of CBD-induced apoptosis among transfected green cells (Figure 6E). These experiments further confirmed a role for MAPK p38 activation in regulation of CBD-induced apoptosis in glioblastoma cells. Possible pro-apoptotic targets of p38 in glioblastoma cells will be investigated in the subsequent experiments of the current study.

### A role of NF-κB suppression for an additional upregulation of CBD-induced apoptosis in glioblastoma cells

**Figure 7:**
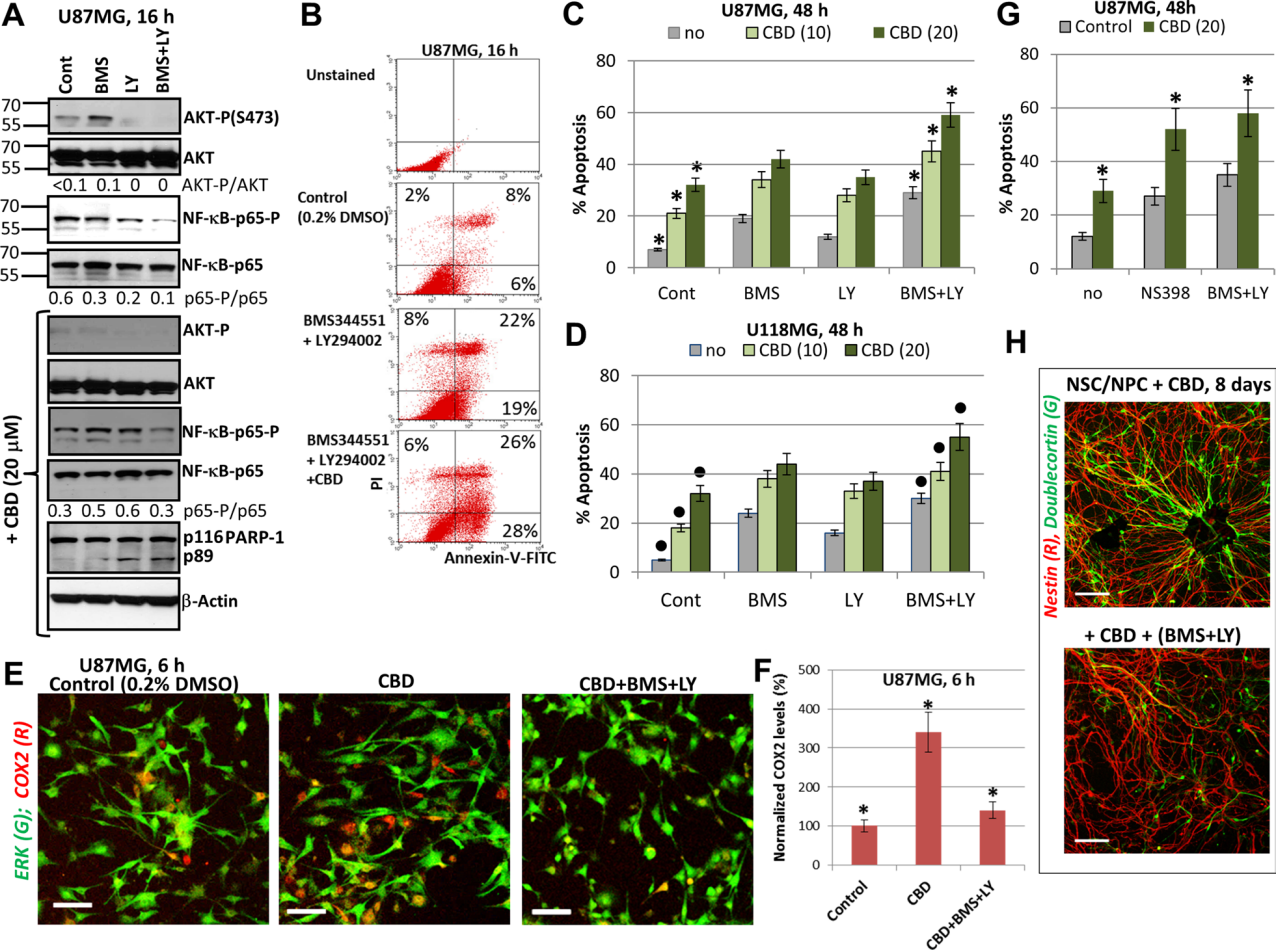
Upregulation of CBD-induced apoptosis using combined inhibition of IKK-NF-κB and PI3K-AKT (**A**) Western blot analysis of signaling proteins in the presence of indicated inhibitors, BMS345541 (20 µM) and LY294002 (50 µM) before and after addition of CBD (20 µM) into cell media. (**B**) The early effects of combined treatment with BMS345541, LY294002 and CBD (20 µM) on induction of apoptosis. Annexin-V-FITC and PI staining with the subsequent FACS analysis was performed. Annexin-V-FITC-positive cells, PI-negative cells are early apoptotic cells while Annexin-V-FITC-positive, PI-positive cells are late apoptotic cells. (**C**, **D**) Effects of small molecule inhibitors on CBD-induced apoptosis in U87MG (panel C) and in U118MG cells (panel D). Cell cycle-apoptosis analysis was performed using PI staining and FACS analysis. The pooled results of four independent experiments after indicated treatments of glioblastoma cells are demonstrated. Error bars represent means ± S.D. *(p* < 0.05, Student’s *t*-test). Stars indicate a significant difference between control CBD-treated U87MG cells and (CBD+inhibitors)-treated cells; black circles indicate the significant differences for U118MG cells. (**E, F**) Upregulation of the number of COX2-expressing U87MG cells 6 h after CBD (20 µM) treatment; this effect was blocked in the presence of BMS345541 and LY294002. Immunostaining with antibodies to ERK1/2 (green) and COX2 (red) and confocal analysis of images were performed. Bar = 50 μm. COX2 levels (normalized to a number of cells) are shown in the panel F. Stars indicate a significant difference. (**G**) Cell cycle-apoptosis analysis was performed 48 h after treatment of U87MG cells by CBD (20 µM) in the presence of COX2 enzymatic inhibitor NS398 (50 µM) or BMS345541 (20 µM) and LY294002 (50 µM). Error bars represent means ± S.D. *(p* < 0.05, Student’s t test). Stars indicate a significant difference between CBD-treated glioblastoma cells and these cells after combined treatment by (CBD and NS398) or (CBD, BMS345541, and LY294002). (**H**) A combination of BMS345541 (20 µM) and LY294002 (50 µM) partially suppressed CBD-induced differentiation of NSC/NPC. Confocal analysis of immunofluorescent images was done using monoclonal Ab against Nestin, an early neuroprogenitor marker (red), and polyclonal Ab against Doublecortin, a neuronal marker (green). Bar = 50 μm.

NF-κB-mediated gene expression controls numerous pro-survival and anti-apoptotic functions in normal and cancer cells. On the other hand, expression of some pro-apoptotic signaling proteins is also under positive control of NF-κB [55–57]. We observed, however, that CBD treatment only modestly affected high basal levels of active NF-κB p65 in GBM cells (see Figure 1A, 1B), suggesting that an additional suppression of NF-κB activity by specific inhibitors might increase CBD-induced apoptotic levels. NF-κB nuclear translocation/activation, as a result of its release from the complex with phosphorylated inhibitor IκB after the proteasome-dependent degradation of IκB [58], required both AKT and IKKβ activities in glioblastoma cells. Indeed, substantial suppression of NF-κB p65-P(S536) and AKT-P(S473) levels by combined treatment of BMS345541 and LY294002 in U87MG cells (Figure 7A) induced significant levels of apoptosis in these cells determined 16 h after treatment by Annexin-V-FITC and PI staining (Figure 7B) or 48 h after treatment determined by PI staining of DNA and cell cycle-apoptosis analysis (Figure 7C). Combined treatment by these inhibitors in the presence of CBD with completely eliminated phospho-AKT levels and notably reduced NF-κB phospho-p65 levels (Figure 7A) additionally increased CBD-induced apoptosis in U87MG (Figure 7B, 7C), as well as in U118MG cells (Figure 7D). Finally, this apoptosis was mediated by caspase-3 (due to the characteristic caspase-3-dependent PARP-1 cleavage; Figure 7A).

NF-κB is the crucial regulator of gene expression of many pro-inflammatory proteins, such as IL6 and COX2. The latter plays a strong anti-apoptotic role for many types of cancers, including glioblastoma [59]. CBD treatment alone or together with irradiation upregulated protein expression levels of COX2 in a number of COX2-positive glioblastoma cells (Figure 7E, 7F). Since the *COX2* promoter activity is controlled by NF-κB and AP1 among other transcription factors, upregulation of AP1 levels (cJUN/ATF2; cJUN/cFOS) appears to increase COX2 expression following CBD exposure. On the other hand, a combination of BMS345541 (10 µM) and LY294002 (50 µM), which suppressed IKK/AKT-mediated activation of NF-κB, further suppressed COX2 protein expression (Figure 7E, 7F) in concert with numerous downstream targets of these pathways. Total ERK levels were relatively stable before and after CBD treatment of U87MG cells and were used for normalization of the COX2 expression levels. NS398 (50 µM), a specific inhibitor of the enzymatic activity of COX2, was known to induce apoptosis in glioblastomas [59]. A combination of NS398 with CBD substantially increased levels of apoptosis and total cell death induced by CBD (20 µM) alone in U87MG cells to levels observed after NF-κB suppression (Figure 7G). Taken together, results obtained demonstrated a protective role of elevated COX2 expression levels and activity, which were induced by CBD in GBM cells, and indicated a possible target pathway to further increase a proapoptotic activity of CBD. Interestingly, triple treatment with CBD, BMS345541 (10 µM) and LY294002 (50 µM) was not toxic for NSC/NPC but abolished stimulating effects of CBD on neuronal differentiation via decreasing expression of Doublecortin, a neuronal marker (Figure 7H).

### Sensitization of glioblastoma to death via death receptor-mediated signaling induced by CBD

**Figure 8:**
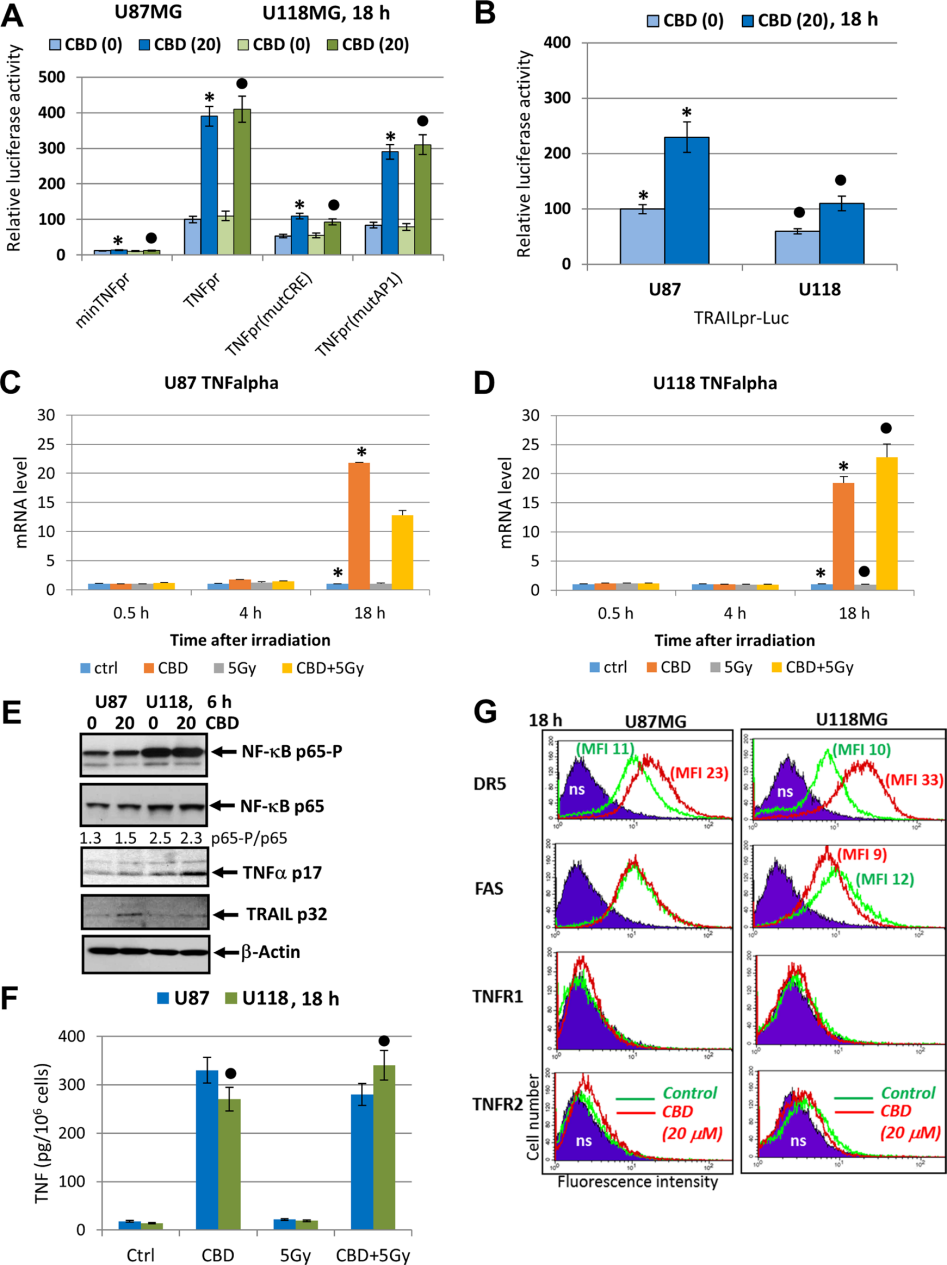
Effects of CBD on TNFα and TRAIL expression and secretion and surface expression of death receptors in glioblastoma cells (**A**) Effects of CBD (20 µM) on TNF-promoter-luciferase reporter activity in transiently transfected glioblastoma cells. “Minimal” -36TNFpr-Luc, -615TNFpr-Luc (wt) and two mutated constructs, -615TNFpr(mutCRE)-Luc and -615TNFpr(mutAP1)-Luc were used. The pooled results of three independent experiments are presented in panel A. Error bars represent means ± S.D. *(p* < 0.05, Student’s *t*-test). Stars and black circles indicate a significant difference in the TNF promoter activity after CBD treatment in U87MG and U118MG cells, respectively. (**B**) Effects of CBD (20 µM) for 6 h on TRAIL-promoter-luciferase activity in transiently transfected glioblastoma cells, 24 h after transfection. (**C**, **D**) Quantitative real-time PCR analysis of the kinetics of TNFα mRNA levels after treatments of U87MG and U118MG cells by CBD (20 µM) and γ-irradiation (5 Gy) alone or in combination. The graphs indicate the fold change of target gene mRNA levels against time-point control after normalized to reference gene (beta-Actin). The pooled results of four independent experiments are presented in panel B. Error bars represent means + S.D. *(p* < 0.05, Student’s *t*-test). Stars indicate a significant difference in mRNA levels between control and CBD-pretreated cells, black circles - between irradiated (5 Gy) and irradiated in the presence of CBD (20 µM) cells. (**E**) Western blot assay of active and total NF-κB p65, TNFα p17 and TRAIL p32 levels 6 h after treatment by CBD in glioma cells. (**F**) TNFα protein levels secreted into the media after indicated treatments of glioblastoma cells were detected by ELISA. (**G**) Effects of CBD treatment on surface expression of death receptors in U87MG and U118MG glioblastoma cells. Surface expression of the main death receptors DR5/TRAIL-R2, FAS, TNFR1 and TNFR2 in U87MG and U118MG cells was determined by immunostaining and the flow cytometry. Cells were treated overnight in presence of 0.1% DMSO (green lines) or 20 µM CBD (red lines); ns–non-specific staining.

Critically important targets of JNK and MAPK p38 signaling, transcription factors cJUN, ATF2 and CREB in concert with NF-κB, activate expression and secretion of proinflammatory cytokines including TNFα and IL1β, as well as death ligands TRAIL and FAS Ligand [49, 60–63]. In non-treated glioma cells, the extrinsic apoptotic pathways are suppressed. Could CBD treatment restore and increase extrinsic apoptotic sensitivity in glioma cells? Since CBD treatment of glioma cells strongly affected JNK pathway, notably MAPK p38 pathway and maintained relatively high basal NF-κB activity, we expected to find CBD-induced upregulation of *TNFα* gene expression. It was a reason first to assess the promoter activity of *TNF* gene after CBD treatment using U87MG cells transiently transfected with the luciferase reporter constructs containing the intact TNF promoter (-615TNFpr-Luc) and its two variants, either with a mutated CRE (-106) site or with a mutated AP1 (-66) site, which lost ability for binding ATF2-JUN and JUN-FOS, respectively, as well as “minimal” -36TNFpr-Luc (Figure 8A) [64, 65]. CBD treatment substantially increased the -615TNF-promoter-directed Luc-reporter activity in both U87MG and U118MG lines (about 3.5-fold). The presence of a mutated CRE site in the promoter decreased levels of the basal Luc activity and strongly down-regulated CBD-induced Luc activity. TNF-promoter-dependent Luc activity notably decreased in the presence of the mutated AP1 site. It demonstrated a possible role of CBD-induced signaling cascades for regulation of *TNF* gene expression in glioblastoma cells (Figure 8A). The human 1.5kbTRAIL promoter contained two NF-κB-binding sites, several TRE/CRE sites (that could bind different combinations of JUN, FOS, ATF2) and GAS site that could bind STAT1/3. These sites and the corresponding transcription factors play a critical role for TRAIL transcription under normal and stress conditions [66–69]. We demonstrated a notable increase of the reporter activity for TRAILpr-Luc in U87MG cells and modest increase in U118MG cells after CBD treatment reflecting a possible role of AP1 activation for regulation of the TRAIL promoter activity in glioma cells (Figure 8B).

The real-time qPCR analysis further demonstrated a dramatic increase in *TNFα* mRNA levels 18 h after treatment with CBD alone in both glioma lines. In U118MG cells, a combination of CBD with γ-irradiation additionally increased *TNFα* mRNA levels, while in U87MG cells, downregulation of CBD-induced levels by γ-irradiation was observed 18 h after treatment (Figure 8C, 8D). Western blot analysis confirmed a notable increase in levels of protein expression of TNFα in U118MG and TRAIL in U87MG cells after CBD treatment (Figure 8E). Secretion of TNFα was also substantially increased in both glioblastoma lines after CBD treatment with a relatively small positive effect of irradiation in combination with CBD for U118MG cells (Figure 8F). Relatively minor expression of endogenous TRAIL was induced after CBD treatment of U87MG cells (Figure 8E).

A remarkable feature of CBD treatment was its effect on surface expression of death receptor DR5/TRAIL-R2 that could be used as an alternative strategy for sensitization of glioblastoma cells to external apoptotic stimulation. Pretreatment by CBD (20 µM) induced significant upregulation of DR5/TRAIL-R2 surface expression in both U87MG and U118MG cells 18 h after exposure (Figure 8G). Of note, suppression of AKT activity after CBD treatment (see Figure 1B), could additionally increase TRAIL-mediated death [70]. CBD treatment, furthermore, modestly increased surface expression of TNFR1 for both cell lines and TNFR2 for U87MG cells, while FAS surface expression was without changes or slightly decreased (Figure 8G).

**Figure 9:**
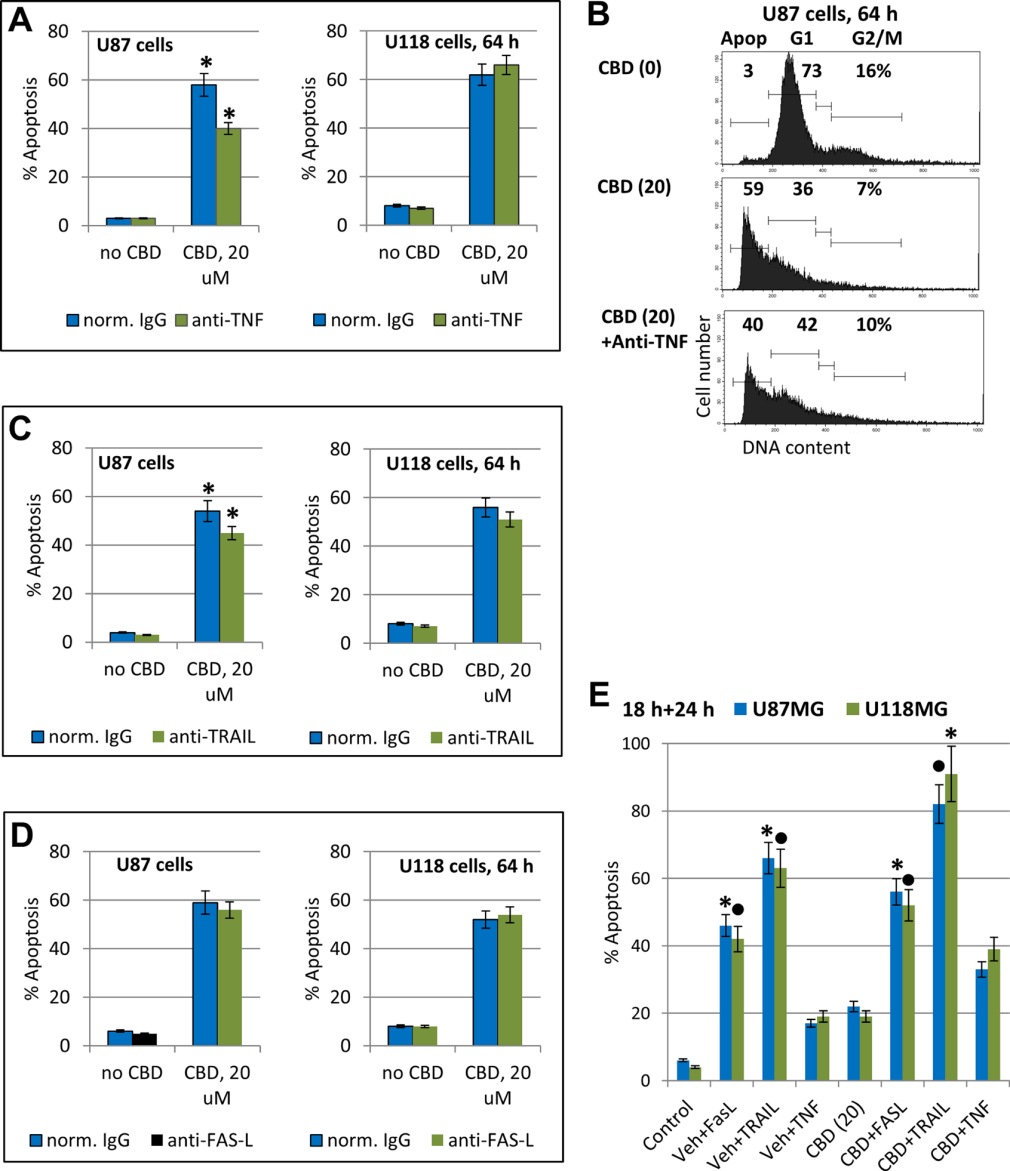
Modulation of apoptosis in glioblastoma cells (**A**–**D**) Effects of anti-TNF inhibitory antibody (5 μg/ml), anti-TRAIL inhibitory antibody (5 μg/ml) or anti-Fas-L inhibitory antibody (5 μg/ml) in the media on CBD-induced apoptosis in U87MG and U118MG cells. Typical experiment of cell cycle apoptosis-analysis with PI-stained nuclei is shown in the panel B. The pooled results of four independent experiments are presented in panels (A–C). Error bars represent means ± S.D. *(p* < 0.05, Student’s *t*-test). Stars indicate a significant difference in CBD-induced apoptotic levels between control and anti-TNF- or anti-TRAIL-treated U87MG cells. (**E**) Effects of CBD (20 µM) pretreatment (18 h) on the subsequent treatment (24 h) of glioma cells with exogenous FasL (50 ng/ml) + CHX (1 µg/ml), TNFα (20 ng/ml) + CHX, and TRAIL (50 ng/ml) + CHX. Levels of apoptosis were determined using cell cycle-apoptosis analysis. Stars and black circles indicated significant differences.

To assess a role of endogenously produced TNFα and TRAIL after CBD treatment of glioblastoma cells, we added inhibitory antibody against TNFα (5 μg/ml) or against TRAIL into the culture media before CBD treatment. Determination of apoptotic levels indicated a notable decrease in CBD-induced apoptosis in the presence of the inhibitory antibody against TNFα in U87MG cells and the absence of effect in U118MG cells (Figure 9A, 9B). Furthermore, CBD-induced apoptosis was modestly decreased by anti-TRAIL Ab in U87MG cells while a decrease in U118MG cells was not significant (Figure 9C). Higher levels of the active NF-κB p65-P(S536) in U118MG cells (Figure 8E) could be one reason for resistance of these cells against TNF-induced apoptosis [71, 72]. We previously observed the endogenous TRAIL secretion by glioma cells in cell cultures [22] that could result in TRAIL-mediated apoptosis via an autocrine/paracrine mechanism in TRAIL-R2/DR5 positive glioblastomas with upregulated surface expression of this death receptor and suppression of phospho-AKT levels after CBD treatment. In contrast, no notable effects on CBD-induced apoptosis in both glioma lines was detected using an inhibitory anti-FAS-L antibody added to the cell media (Figure 9D) correlating with a non-essential role of the endogenous FAS-L in regulation of CBD-induced death.

Among three recombinant death ligands, TNFα, FasL, and TRAIL, exogenous TRAIL in combination with cycloheximide (CHX), 1mg/ml, a classical accelerator of apoptosis, induced higher levels of apoptosis in CBD-pretreated U87MG and U118MG cells (Figure 9E). The effective delivery of death ligand TRAIL to glioblastoma cells *in vivo* using NSC as a vehicle with overexpression and secretion of TRAIL [73] could probably be used in combination with CBD pre-treatment for a further increase in efficacy of TRAIL-mediated apoptosis in glioblastoma cells.

### Regulation of cytokine gene expression by CBD alone or in combination with γ-irradiation

**Figure 10:**
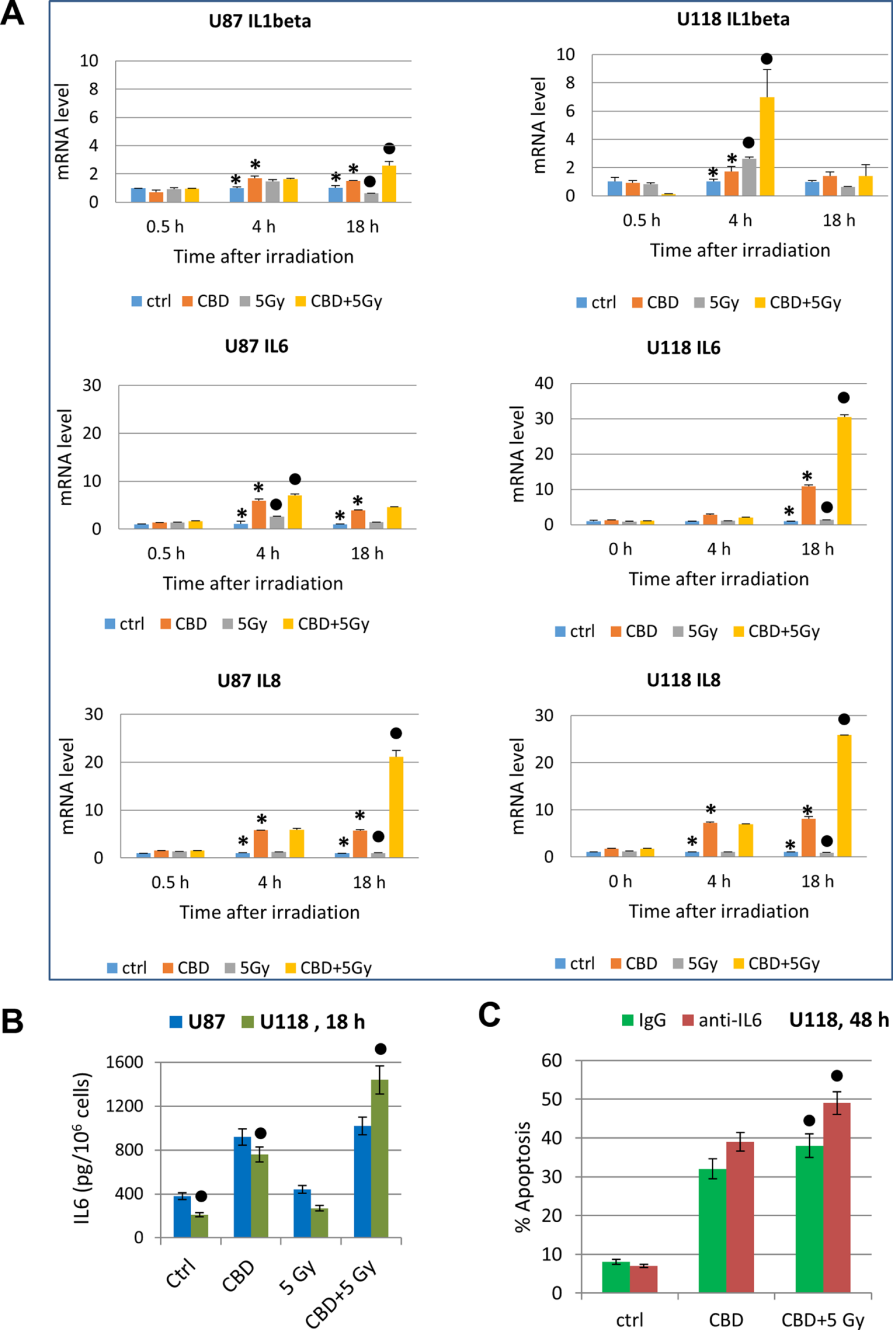
Cytokine gene expression in glioblastoma cells determined by quantitative real-time PCR **(A)** Quantitative real-time PCR analysis of IL1β, IL6 and IL8 mRNA levels after treatments of U87MG and U118MG cells by CBD (20 µM) and γ-irradiation (5 Gy) alone or in combination. The graphs indicate the fold change of target gene mRNA levels against time-point control after normalized to reference gene (beta-Actin). The pooled results of four independent experiments are presented. Error bars represent means ± S.D. *(p* < 0.05, Student’s *t*-test). Stars indicate a significant difference in mRNA levels between control and CBD-pretreated cells, black circles - between irradiated (5 Gy) and irradiated in the presence of CBD (20 µM) cells. (**B**) IL6 protein levels secreted into the media after indicated treatments of glioblastoma cells were detected by ELISA. Black circles indicate significant differences in IL6 secretion after treatment of U118MG cells CBD (20 µM) alone or in combination with irradiation (5 Gy). (**C**) Effects of anti-IL6 inhibitory antibody (5 μg/ml) in the media on apoptosis induced by CBD (20 µM) alone or combined treatment of CBD and γ-irradiation of U87MG cells. Black circles indicate a significant upregulation of apoptosis after combined treatment of U118MG cells in the presence of anti-IL6 Ab.

Glioblastomas are known as active producers of numerous pro-inflammatory cytokines, including besides TNFα, cytokines IL1β, IL6 and IL8, and growth factors (such as TGFβ) that affect via paracrine/autocrine signaling the cancer cells themselves and actively participate in intercellular communication with cancer microenvironment including tumor infiltrating lymphocytes [74]. We examined effects of CBD and γ-irradiation, alone or in combination, on gene expression of these cytokines in U87MG and U118 cells using real-time qPCR (Figure 10). CBD (20 µM) alone and, especially, in combination with γ-irradiation (5 Gy), induced a strong increase IL1β and IL6 after combined treatment (4 h and 18 h, respectively) in U118MG cells and less pronounced changes in U87MG cells, a substantial increase in gene expression of IL8 in both cell lines (Figure 10A); and minor changes in already high TGFβ levels in both lines (data not shown). Protein secretion of IL6 into the culture media was correlated with level of gene expression (Figure 10B). Hence, CBD in combination with γ-irradiation, via regulation of pro-inflammatory signaling in glioblastoma could affect a balance between survival and death of cancer cells in stress conditions. We suggested that prolonged expression of a high level of IL6 after combined treatment of U118MG cells might result in decreased levels of apoptosis in these cells. Indeed, addition of anti-IL6 inhibitory Ab (5 µg/ml) to the cell media significantly increased levels of apoptosis in glioma cells after treatment with CBD alone or in combination with γ-irradiation (Figure 10C).

### Effects of antagonists of CB1 and CB2 receptors on CBD-mediated signaling cascades in glioblastoma lines

**Figure 11:**
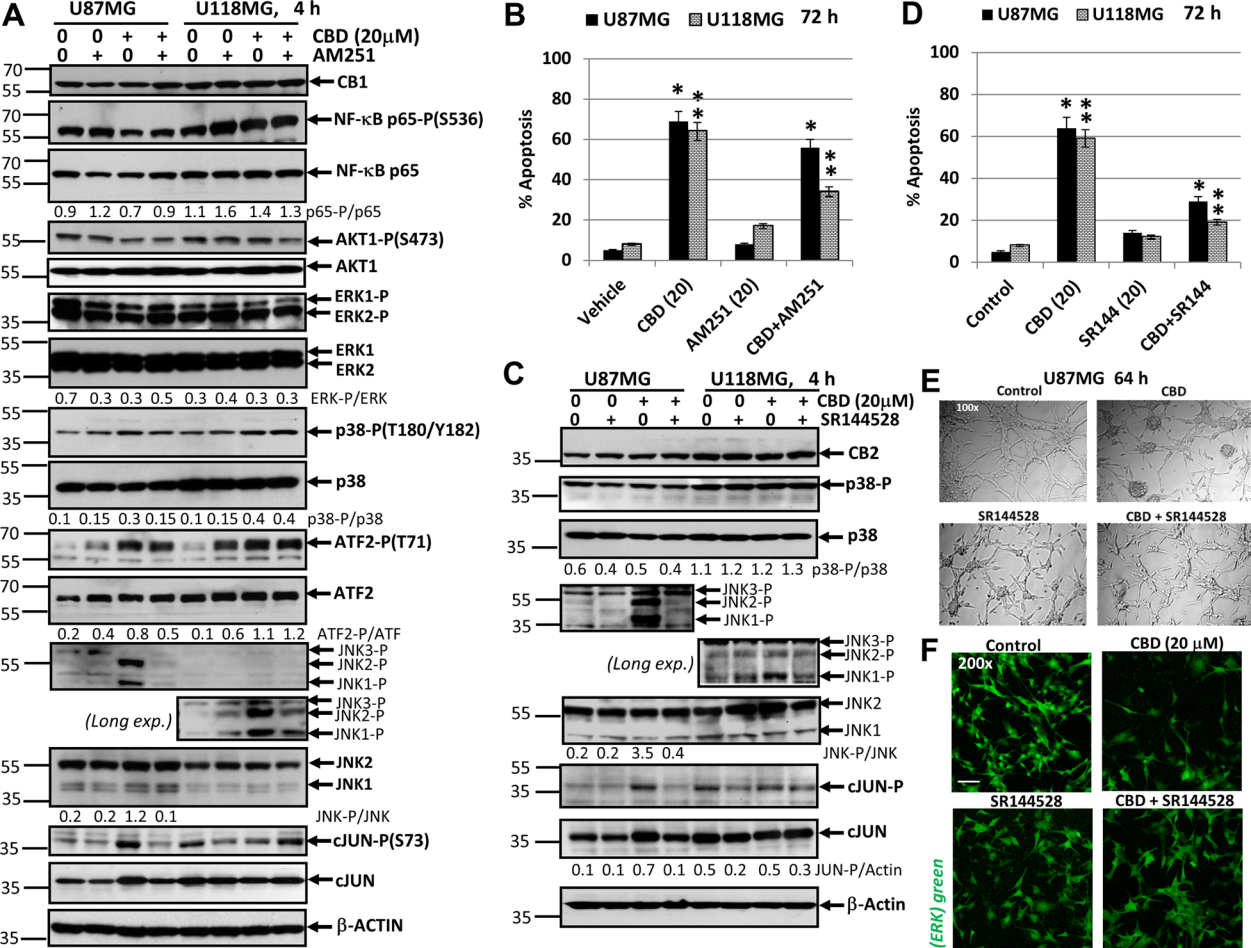
Effects of cannabidiol (CBD) alone or in combination with (i) AM251, an antagonist of CB1-receptor mediated signaling cascades, (ii) SR144528, an antagonist of CB2-receptor mediated signaling cascades, on main signaling pathways and apoptosis in U87MG and U118MG glioblastoma cell lines (**A**) Effects of AM251 (20 µM), on CBD-induced (20 µM) changes in main signaling pathways were determined in glioblastoma cell lines using Western blot analysis. JNK-P detection is shown on two subpanels with normal (for U87MG) and prolonged exposure (for U118MG) of X-ray film during ECL. Quantitative densitometry of protein bands was performed. (**B**) Effects of AM251 (20 µM), on CBD-induced (20 µM) apoptosis in glioblastoma lines. Vehicle contains 0.2% DMSO. Cell cycle-apoptosis analysis was performed using PI staining DNA and the flow cytometry. The pooled results of four independent experiments are presented on the upper panel. Error bars represent means ± S.D. *(p* < 0.05, Student’s *t*-test). Stars indicate a significant difference between (CBD+Vehicle)- and (CBD+AM251)-treated U87MG cells; double stars -- between (CBD+Vehicle)- and (CBD+AM251)-treated U118MG cells. (**C**) Effects of SR144528 (20 µM), on CBD-induced (20 µM) changes in main signaling pathways were determined in glioblastoma cell lines using Western blot analysis. Quantitative densitometry of protein bands was performed. (**D**) Effects of SR144528 (20 µM), on CBD-induced (20 µM) apoptosis was determined 72 h after treatment. (**E**) Phase-contrast microscopy of U87MG cells 64 h after indicated treatment: control, DMSO 0.2%; CBD 20 µM; SR144528 20 µM; and combination of (CBD+SR144528). Typical images are shown. (**F**) Immunostaining total ERK (green) and fluorescent microscopy of U87MG cells 64 h after indicated treatment. Typical images are shown. Bar = 50 μm.

U87MG and U118MG glioblastoma cells exhibit high protein expression of cannabinoid receptors, CB1 and CB2 (Figure 11A, 11C) and a well pronounced dose-dependent apoptotic response to CBD treatment. In contrast to ∆THC and endocannabinoids, CBD only slightly interacts with CB1 and CB2 receptors but after penetration into glioma cells it induced oxidative stress [40, 75].

The problem of CB1/2 receptor interfering with CBD-mediated signaling is not still completely resolved. In spite of suggestion about CB1/2 independent or almost independent mechanism of CBD signaling, mutual interference between CB1/2 receptor- and CBD signaling was observed [48, 76, 77]. Based on this suggestion, we evaluated possible downstream effects of the antagonists of CB1- or CB2-receptor signaling on CBD-mediated signaling cascades in glioblastomas. For this purpose, CBD (20 µM) was used alone or together with a CB1 receptor antagonist (inverse agonist) AM251 (20 µM) or with a CB2 receptor antagonist (inverse agonist) SR144528 (20 µM) (Figure 11) in U87MG and U118MG cells for 4h exposure. AM251 diluted in DMSO was added to cell cultures 15 min before CBD. DMSO (at final concentration 0.2%) was used as a vehicle. We observed moderate variations of the high basal levels of phospho-p65 NF-κB, phospho-AKT and phospho-ERK1/2 4 h after CBD treatment detected in both glioblastoma lines; these active levels were insensitive to co-treatment with AM251 (Figure 11A). Furthermore, CBD-mediated increase in phospho-MAPK p38 levels, as well as upregulation of phospho-ATF2 levels were not notably affected by AM251 in U87MG and U118MG cells. The most remarkable effect of AM251 was suppression of CBD-induced of JNK1/2 phosphorylation in U87MG (Figure 11A). It could also be observed in U118MG cells after prolonged exposure of X-ray film during ECL (Figure 11A). Upregulation of phospho-(Ser73)-cJUN level by CBD was partially suppressed in the presence of AM251 in U87MG cells; it was well correlated with phospho-JNK1/2 levels in U87MG cells (Figure 11A). Due to a deficiency of active JNK1/2 in U118MG cells, cJUN could be additionally phosphorylated by a different kinase, such as ERK1/2, and was not decreased by AM251 in these cells. Hence, AM251 effectively suppressed CBD-induced JNK1/2 activation in U87MG and partially in U118MG cells in concert with relatively minor effects on several signaling pathways. These effects of AM251 may reflect prevention of oxidative stress, which could be induced by CBD treatment.

Next, we addressed a question regarding probable effects of AM251 on CBD-induced apoptosis. Quite surprisingly, AM251 (20 µM) more efficiently suppressed CBD-induced apoptosis in U118MG compared to U87MG cells 72 h after treatment (Figure 11B). Data obtained were correlated with a minor proapoptotic role for JNK-cJUN in CBD-induced apoptosis but did not reveal critical targets for AM251-dependent anti-apoptotic activity in several signaling pathways in glioblastoma cells. Immunostaining with Ab to total ERK and fluorescent microscopy also demonstrated a non-effective protection of AM251 against CBD-induced U87MG cell death (data not shown).

On the other hand, a combination of CBD and SR144528 (20 µM), an antagonist (inverse agonist) of CB2 receptor, did not demonstrate notable effects on NF-κB p65-P, ERK-P, AKT-P (data not shown) and MAPK p38-P levels in U87MG and U118MG cells (Figure 11C). The prominent effect of SR144528 in combination with CBD was again a suppression of CBD-induced activation of JNK1/2 in U87MG cells (Figure 11C). This effect was only modestly pronounced in U118MG cells and could be detected after prolonged exposure of X-ray film during ECL. It was accompanied by the corresponding downregulation in CBD-induced phospho-cJUN levels in U87MG cells but not in U118MG cells (Figure 11C).

SR144528 effectively decreased CBD-induced apoptosis at relatively early phase that was detected with Annexin-V-FITC and PI staining in both GBM lines 16 h after treatment (data not shown) and substantially downregulated total apoptotic (the sub-G1) levels 72 h after treatment determined by cell cycle and apoptosis analysis using PI-staining DNA and the flow cytometry (Figure 11D). We also observed, using the phase contrast microscopy and immunostaining for ERK-positive cells, that the presence of SR144528 in the culture media substantially reduced CBD-induced damage in the GBM cell cultures and decreased the levels of GBM cell death (Figure 11E, 11F). The similar protective effects of SR144528 were observed in CBD-treated U87MG cells (data not shown). In general, signaling effects of CB1 and CB2 receptor antagonists appear to be strongly linked with effects on CBD-induced oxidative stress. Furthermore, our observations indicated on more efficient suppression of CBD-induced apoptosis by antagonist of CB2 receptor signaling, compared to AM251, in glioblastoma cells. A similar effect of a CB2 receptor antagonist on suppression of CBD-induced apoptosis in glioblastoma cells was previously observed [48].

**Figure 12:**
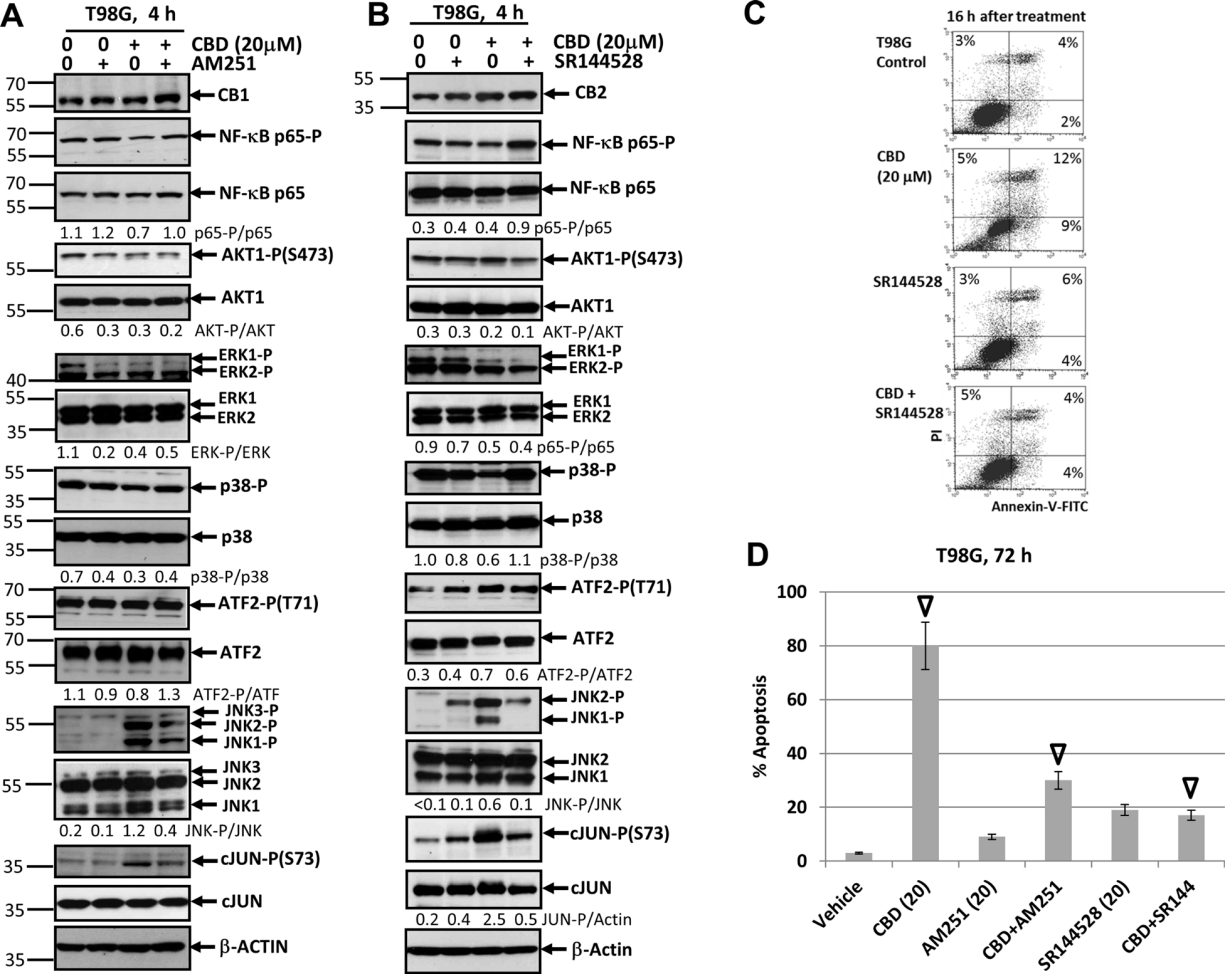
Effects of cannabidiol (CBD) alone or in combination with (i) AM251, an antagonist of CB1-receptor mediated signaling cascades, (ii) SR144528, an antagonist of CB2-receptor mediated signaling cascades, on main signaling pathways and apoptosis in T98G glioblastoma cell lines (**A**, **B**) Effects of AM251 (20 µM) or SR144528 (20 µM) on CBD-induced (20 µM) changes in main signaling pathways were determined in T98G cells Western blot analysis. Quantitative densitometry of protein bands was performed. (**C**) Effects of SR144528 (20 µM), on CBD-induced (20 µM) apoptosis 16 h after treatment. Vehicle contains 0.2% DMSO. Annexin-V-FITC and PI staining was performed and followed by FACS analysis. (**D**) Effects of AM251 (20 µM) or SR144528 (20 µM) on CBD-induced (20 µM) apoptosis in T98G cells. Vehicle contains 0.2% DMSO. Cell cycle-apoptosis analysis was performed using PI staining DNA and the flow cytometry. The pooled results of four independent experiments are presented on the upper panel. Error bars represent means ± S.D. *(p* < 0.05, Student’s *t*-test). Open arrows indicate a significant difference between (CBD+Vehicle), (CBD+AM251)- and (CBD+SR144528)-treated T98G cells.

To further extend our study, we elucidated effects of CB1/2 receptor antagonists on CBD-induced apoptosis in the T98G human glioblastoma line, which was also CB1- and CB2-receptor positive and contained mutated p53 and PTEN (Figure 12). The presence of AM251 or SR144528 did not significantly affect the basal or CBD-mediated AKT-P and NF-κB-P levels in T98G cells. There was also a tendency for downregulation of ERK-P 4 h after CBD treatment that was not restored after co-treatment with AM251 and SR144528. The basal levels of MAPK p38-P were high and CBD alone might have a moderate negative effect on p38-P level in T98G cells (Figure 12B). A dramatic upregulation JNK1/2-P activity accompanied by increased phosphorylation of its major downstream target, cJUN, was also a characteristic feature of CBD treatment in this line that was efficiently suppressed by SR144528 co-treatment and less efficiently by AM251 (Figure 12A, 12B). Apoptotic effects of CBD were well pronounced in T98G cells using both the detection with Annexin-V-FITC + PI 16 h after treatment (Figure 12C) and cell cycle-apoptosis analysis 72 h after treatment. AM251 and, especially SR144528, effectively suppressed the apoptotic commitment (Figure 12D).

**Figure 13:**
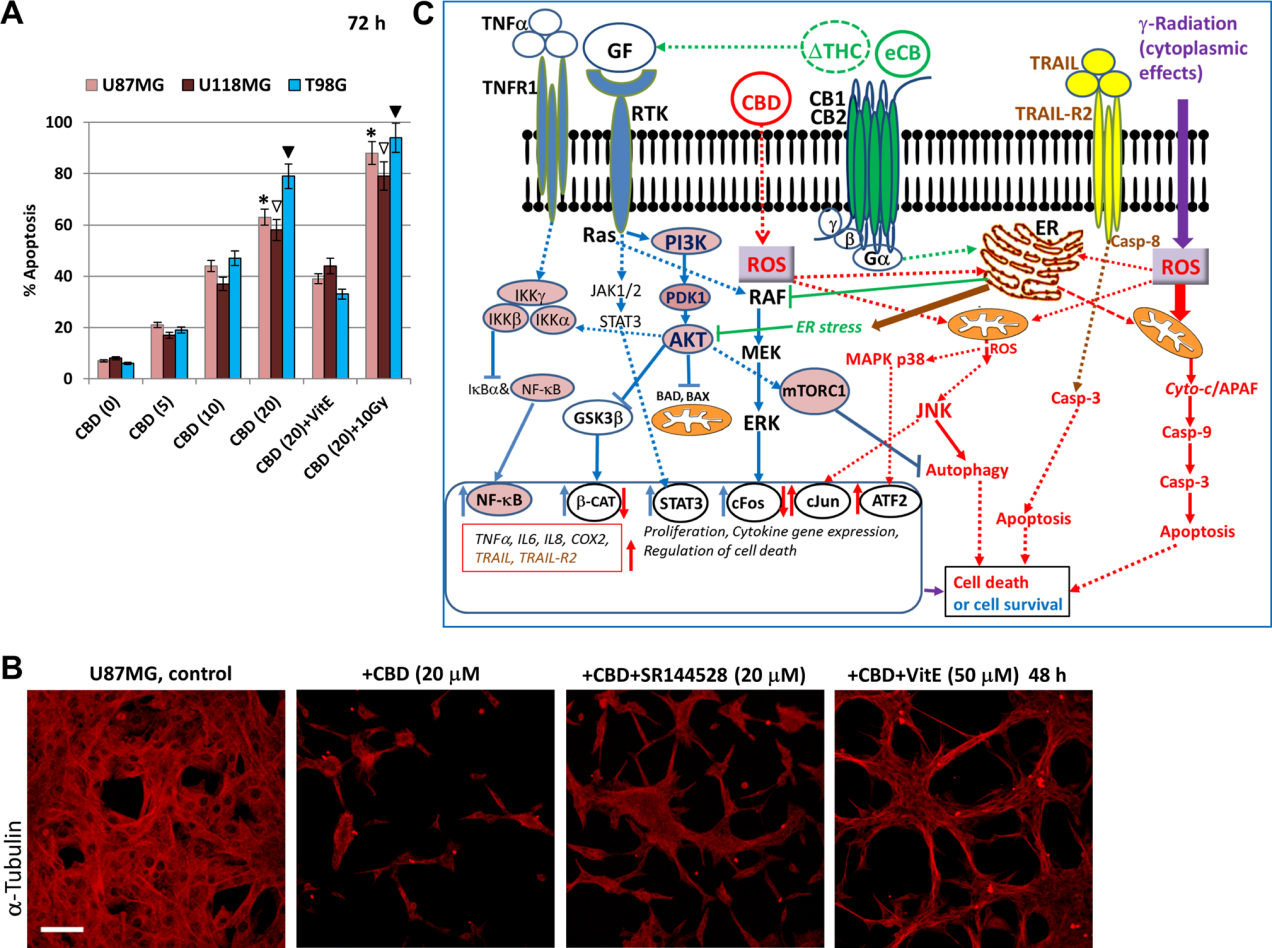
Effects of cannabinoids and γ-irradiation on cell signaling pathways and regulation of apoptosis in glioblastoma cells (**A**) Dose-dependent effects of CBD alone, in combination with VitE (50 µM), or in combination with γ-irradiation (10 Gy) on apoptosis of human glioblastoma cells, which was determined by PI staining DNA and the flow cytometry. The pooled results of four independent experiments are presented on the upper panel. Error bars represent means + S.D. *(p* < 0.05, Student’s *t*-test). Stars, open arrows and black arrows indicate significant differences between CBD-treated and (CBD+10Gy) treated U87MG, U118MG and T98G cells, respectively. (**B**) CBD-induced cell death was partially suppressed in the presence of SR144528 (20 µM), a CB2 receptor antagonist, or Vitamin E. U87MG cells were immunostained using anti-β-tubulin monoclonal Ab. Bar = 50 µm. (**C**) Regulation of signaling pathways and apoptosis in the presence of cannabinoids. ∆THC and endocannabinoids (eCB) induce signaling cascades through CB1 and CB2 receptors. ∆THC could also affect growth factor receptors (GFR) signaling. CB1/2 receptors coupling to Gi signaling assemble is associated with activation of RAF-MEK-ERK pathway in the normal neural and glial cells, as well as in glioma cells. Activation of the PI3K/Akt-mTORC1 pathway by GFR with tyrosine kinase activity (RTK) might be negatively affected by CB1 receptor-dependent activation of ERK. RTK signaling activates also JAK/STAT3 pathway. ∆THC-induced activation of CB1 and CB2 receptors upregulates ceramide synthesis in the endoplasmic reticulum (ER) and induces ER-stress, which suppresses/ downregulates RTK-induced AKT and ERK activation in glioma cells. It further suppresses mammalian target of rapamycin complex-1 (mTORC1) activity accompanied by suppression of the general translation but upregulation of autophagy. In contrast, cannabidiol (CBD) may directly penetrate into the cells without binding CB1/2 receptors. In the cells, CBD dramatically upregulates ROS production and ROS-dependent network, including activation of JNK. MAPK p38 could be activated by CBD in both ROS-dependent and ROS-independent mechanisms. These signaling pathways further regulate mitophagy, autophagy and the mitochondrial apoptotic pathway in glioblastoma cells, synergizing with γ-irradiation-induced cell death. CB1/2 receptors still could control CBD-induced signaling. TNFα /TNFR1 signaling might play a dual role via regulation of both survival functions (via NF-κB) and apoptotic or necroptotic functions. CBD treatment in combination with γ-radiation strongly affects TNF and TNF-R1 expression with the subsequent induction of proinflammatory cytokine (IL6 and IL8) expression and secretion. CBD could upregulate production of TRAIL and surface expression of TRAIL-R2 restoring external apoptotic signaling in glioblastoma cells.

In general, we demonstrated cell-specific quantitative differences in CBD-induced regulation of some major signaling proteins in U87MG, U118MG and T89G glioblastoma lines. A downstream cross-talk of CBD signaling (such as upregulation of phospho-JNK) with antagonists of CB1- and CB2-dependent signaling resulted in suppression of this upregulation in two glioblastoma lines. This quite remarkable phenomenon, which had only a moderate effect on CBD-induced apoptosis, needs an additional investigation, as well as elucidation of additional targets that protect glioma cells against CBD-induced apoptosis. Interestingly, combined activation of CBD-signaling and ∆THC-CB1/2 dependent signaling exhibited synergistic effects and induced high levels of apoptosis in glioblastomas [29, 47]. Accelerating effects of γ-irradiation on CBD-induced apoptosis were observed in the present study for three glioblastoma lines (Figure 13A). On the other hand, lipid-soluble antioxidant, such as vitamin E, might partially inhibit CBD-induced oxidative stress and apoptosis or total cell death in glioblastoma cells (Figure 13A and 13B). Activation of both JNK and MAPK p38 and their downstream targets, such as TRAIL and its receptor TRAIL-R2, seems especially important for mediation of CBD-induced apoptosis in gliomas through the external apoptotic signaling mechanism.

## DISCUSSION

The mechanism of natural surveillance and killing of cancer cells based on the enhancement of the immune response has been successfully used in immunotherapy, due to the introduction of the immune checkpoint inhibitors [78–80]. There are, however, significant problems to effectively apply immunotherapy for brain tumor treatment, due to the blood-brain barrier. An alternative and complementary possibility for cancer treatment is an intensive elucidation of cell signaling receptors, which regulate signal-dependent gene expression and are present at increased levels in cancer cells. CB1 is the most abundant cell surface receptor in the brain; CB2 receptor is also widely expressed on glial cells [77]. Both receptors by binding endocannabinoids activate signaling cascades, which control proliferation, survival and other normal functions.

Numerous investigations highlighted a surprising role for cannabinoids in brain tumors: ∆THC via activation of the corresponding CB1 and CB2 receptors could induce cell death in human glioblastoma and in some other types of cancer [28, 32, 39]. We have focused for this study on the signaling pathways that could regulate killing effects of cannabidiol (CBD), a non-toxic cannabinoid without psychogenic activity and with low affinity for CB1 and CB2 receptors. In spite of CB1/2-receptor independent initiation of CBD signaling pathways, there was a downstream cross-talk of these signaling pathways with CB1/CB2-mediated signaling, as we demonstrated in the present study using specific antagonists (inverse agonists) of CB1- and CB2 receptors.

Characteristic features of CBD-induced signaling in glioblastomas represent the consequences of oxidative stress [40] and include the early induction/ upregulation of JNK-cJUN and modulation of MAPK p38-ATF2 signaling pathways and the late downregulation of the levels of an active phosphorylated form of AKT and ERK involved in regulation of cell proliferation and survival (Figure 13C). In general, strong suppression of AKT1-mTOR and a partial suppression of ERK pathways, in concert with substantial upregulation of JNK activation, the high basal or induced MAPK p38 activity, and very active p53-BAX, may promote autophagy/apoptotic commitment in glioblastoma cells, as was previously investigated [39, 41, 81, 82]. Taken together, these changes in cell signaling dramatically misbalanced the regulation of autophagy, cell survival, and death [52] (see Figure 13C). An important observation previously reported for normal young and mature astrocytes and confirmed in our investigation for NSC/NPC was the absence of pro-apoptotic signaling by CBD in normal neural cells [32, 39].

We demonstrated three additional approaches to further increase killing efficiency of CBD in glioblastomas. The first quite traditional approach was based on a general suppression of PI3K-AKT and IKK-NF-κB signaling pathways. A universal role of upregulation of PI3K-AKT and IKK-NF-κB signaling pathways in cancer cells (based on both genetic and epigenetic mechanisms) has been well established in many types of tumors, including glioblastoma. It also increased resistance against the lethal effects of radiation, chemotherapy or oxidative stress [83, 84]. Consequently, suppression of PI3K-AKT and NF-κB signaling pathways was used for sensitization of human glioblastomas to chemotherapy or death-receptor mediated apoptosis [70, 85, 86]. CBD treatment that was accompanied by the late inhibition of AKT pathway did not notably decrease NF-κB activity. Combined treatment with small molecule inhibitors of IKKβ and PI3K-AKT that was necessary for additional suppression of NF-κB activation substantially increased CBD-induced apoptosis in three glioblastoma lines, as observed in the current study.

An MAP3K, ASK1, was active in glioblastoma lines used in the present study (our non-published data) and could be further upregulated by different types of stress including oxidative stress, endoplasmic reticulum (ER) stress, and calcium influx [87, 88]. Since cannabinoids are involved in the initiation of both oxidative stress [40] and ER stress [44] in glioblastoma, they could possibly target ASK1 activity (modulating its phosphorylation, the subsequent activation, and final degradation) and then its downstream targets, MAPK p38 and JNK. Upregulation of ASK1 activity in U87MG cells after CBD treatment and γ-irradiation could possibly be correlated with downstream p38-ATF2 and JNK activation. This challenging problem for a potential role of ASK1 in CBD-induced signaling in glioblastoma cells needs additional investigation.

The second approach was based on a combination of CBD treatment and subsequent γ-irradiation resulting in the effective induction of apoptosis in three glioblastoma lines using relatively high doses of γ-irradiation and CBD. The current external beam radiation therapy of glioblastoma at very high doses (56–60 Gy) alone or in combination with chemotherapy results in the mitotic catastrophe, arresting cell proliferation and, finally, in necrotic death of tumor cells, as well as of targeted normal cells in the areas of treatment. A possibility to decrease radiation doses in the presence of alternative inducers of cell death, such as CBD and ∆THC, alone or especially in combination looks as a promising alternative to the standard glioblastoma treatment, based on the recent animal experiments with the triple combination, CBD, ∆THC and γ-irradiation [29]. Interesting one aspect of combined treatment by CBD and γ-irradiation was the order of the treatment: first CBD then irradiation; delay of CBD treatment decreased apoptosis. Kinetics of CBD-induced expression and secretion of cytokines (both prosurvival and proapoptotic) appears to significantly affect cell death regulation and synergy between CBD and irradiation. So, suppression of CBD-induced IL6 autocrine/paracrine stimulation of the protective signaling (Figure 10C) might be involved in upregulation of apoptosis induced by combined treatment.

The third approach for sensitization of glioblastoma cells to death via increased surface expression of death receptors including TNF receptors and TRAIL-R2/DR5 based on the corresponding actions of CBD was also demonstrated in the current study (see Figures 8 and 9). Interestingly, CBD-induced down-regulation of AKT and ERK1/2 activities may result in down-regulation of FOXO3A phosphorylation and upregulation of its nuclear translocation/ nuclear function that further upregulated the endogenous TRAIL expression, probably, in concert with AP1, ATF2 and NF-κB. We indeed observed a CBD-induced increase of TRAIL protein levels in U87MG glioblastoma cells (Figure 1B and 8E). Such a regulatory mechanism was also described for TIC10, a pro-apoptotic small molecule inducer, which was found after an intensive screening of the chemical libraries [89]. Furthermore, TIC10 could trigger cytotoxicity in solid tumor cells by inducing a stress response [90].

Multiple cytokine gene expression and secretion induced by combination of CBD and γ-irradiation, as we observed in the present study, additionally complicated a roadmap of signaling events in glioblastomas. There is a direct connection between TGFβ, IL6-STAT3, IL1β-NF-κB, TNFα-NF-κB and JNK-AP1 pathways and *COX2* gene expression, PGE2 production and regulation of stability of exogenous cannabinoids and endocannabinoids [91–93]. We found in the present study an additional functional combination of irradiation and CBD-dependent modulation of cell signaling pathways that further permit an increase of efficiency of glioblastoma treatment with some radioprotective effect for NSC/NPC and, probably, for neurons. Since COX2 oxygenates and inactivates both exogenous and endogenous cannabinoids, we suggested that suppression of COX2 activity by a specific enzymatic inhibitor [59] could substantially activate of cannabinoid signaling cascades [92] and cannabidiol-induced apoptosis. We indeed observed such enhanced CBD-induced apoptosis in the present study (see Figure 7). In general, our study provides additional possibilities to enhance clinical usage of cannabidiol for brain cancer treatment and indicate the molecular targets for further improving efficiency of treatment.

## MATERIALS AND METHODS

### Reagents

PI3K inhibitor LY294002, IKK inhibitor BMS345541, MEK inhibitor U0126, MAPK p38 inhibitor SB203580 and JNK inhibitor SP600125 were purchased from Calbiochem (La Jolla, CA, USA). Human soluble Enzo Killer-TRAIL (recombinant #ALX-201-073-C020), *Super*-Fas-Ligand (recombinant; #ALX-522-020) and anti-human TRAIL Ab (#ALX-210-732) were purchased from Enzo Life Sciences (San Diego, CA, USA). Recombinant human TNFα (#210-TA-010) and anti-human TNFα mAb (#MAB610-100) were obtained from R&D Systems (Minneapolis, MN). Anti-human IL6 mAb (#MAB2061R) were purchased from R&D Systems (Minneapolis, MN). Cannabidiol (exempt preparation; #90081); AM251 (#71670); SR144528 (#192703-06-3) and NS398 (#70590) were obtained from Cayman Chemical (Ann Arbor, MI).

### Human embryonic neural stem cells (NSC) in culture

Cryopreserved human embryonic neural stem cells (NSC) were obtained from Gibco/Life Technologies (Carlsbad, CA, USA) as a commercially available product (N7800-200). The cells were derived from NIH approved H9 (WA09) human embryonic stem cells. The cells were plated in 6-well culture plates coated with fibronectin and incubated at 37°C in complete growth medium NSC/SFM, which contained serum-free DMEM/F12 supplemented with 2 mM GlutaMAX, bFGF (20 ng/ml), EGF (20 ng/ml) and StemPRO neural supplement (2%). All reagents were obtained from Gibco/Life Technologies (Carlsbad, CA, USA).

Neural stem cells were plated on polyornithine- and laminin-coated 6-well plates, which contained similarly coated cover slips, in complete NSC/SFM. After 2 days, neuronal differentiation was initiated by neuronal differentiation media, which contains Neurobasal medium, B-27 Serum-free supplement (2%) and 2 mM GlutaMAX (Gibco/Life Technologies). The medium was changed every two days. A neuronal phenotype was confirmed using immunofluorescence detection 8–10 days after initiation of differentiation.

### Glioblastoma cell lines

Three human glioblastoma lines, which were used in the present study, were obtained from the ATCC: i) U87MG (HTB-14): very tumorigenic, highly invasive, highly rearranged hypodiploid with several hundred mutations, *TP53wt, PTENmut;* ii) U118MG (HTB-15): tumorigenic, moderate invasive; genome average copy number 2.2, several hundred mutations, *TP53mut, PTENmut* iii) T98G (CRL-1690): non-tumorigenic; moderate invasive, highly rearranged hypopentaploid/ hypohexaploid, several hundred mutations, *TP53mut, PTENmut*) [85, 94]. There are some contradictions about the origin and maintenance of the U87MG (HTB-14) glioma cell line in the ATCC [95]; however, they do not interfere the results of the present study (http://cansar.icr.ac.uk/cansar/search_results/158086/; https://portals.broadinstitute.org/ccle_legacy/home). All glioblastoma lines were cultured as described in DMEM with 10% FBS and 1% pyruvate [96].

### Immunocytochemistry analysis

Cells were fixed with 4% paraformaldehyde in PBS for 60 min. Immunochemical staining was performed using standard protocols. NSC/NPC and young neurons were stained for the undifferentiated NSC marker, Nestin (using mAb #MAB5326 from Millipore, Temecula, CA, USA) and for the neuronal marker, Doublecortin (using Ab #4604 from Cell Signaling, Danvers, MA, USA). Glioblastoma cells were stained for ERK1/2 (Ab #9102 from Cell Signaling), COX2 (mAb #160112 from Cayman Chemical) and α-Tubulin (mAb #T5168 from “Sigma”). The secondary Abs were Alexa Fluor 594 goat anti-mouse IgG and Alexa Fluor 488 goat anti-rabbit IgG from Molecular Probes/Life Technologies (Carlsbad, CA, USA). A laser scanning confocal microscope (Nikon TE 2000 with EZ-C1 software, Tokyo, Japan) was used for immunofluorescence image analysis.

### Irradiation procedures

To determine sensitivity to γ-rays, plated NSC, astrocytes and glioblastoma cells were exposed to radiation from a Gammacell 40 ^137^Cs irradiator (dose rate, 0.82 Gy/min) at Columbia University. Six to 48 h after irradiation, cells were stained with PI and analyzed by flow cytometry for cell cycle-apoptosis studies.

### FACS analysis of TRAIL-R2/DR5, TRAIL-R1/DR4, FAS and TNF-R levels

Surface levels of TRAIL-R2/DR5 (PE anti-human DR5 Ab, eBioscience #12-9908), TRAIL-R1/DR4 (PE anti-human DR4, eBioscience #12-6644); FAS/CD95 (PE anti-human CD95 mAb, BD Pharmingen #555674) , TNF-R1 (PE anti-human TNFR1 mAb, R&D #FAB225P) and TNF-R2 (PE Rat anti-human CD120b #552418, BD Pharmingen) on human glioblastoma cell lines were determined by staining with the corresponding Abs and subsequent flow cytometry. A FACS Calibur flow cytometer (Becton Dickinson, Mountain View, CA, USA) and the CellQuest program were used to perform flow cytometric analysis. All experiments were independently repeated 3–5 times.

### Cell death studies

For induction of apoptosis, cells were exposed to cannabidiol and to γ-irradiation (5–10 Gy) alone or in combination. In some experiments, small molecule inhibitors of cell signaling pathways were additionally used. Furthermore, apoptosis was induced TRAIL, TNFα, FasL, and CHX alone or in combination. Apoptosis levels (% of apoptotic nuclei) in cells after fixation and permeabilization by 70% ethanol were assessed by propidium iodide (PI) staining and quantifying the percentage of hypodiploid nuclei (pre-G1) using FACS analysis. Alternatively, staining of fresh cells by Annexin-V-FITC + PI and quantifying the percentage of Annexin-V-FITC-positive, PI-negative cells (the early apoptotic), Annexin-V-FITC-positive, PI-positive cells (the late apoptotic) and Annexin-V-FITC-negative, PI-positive cells (the secondary necrotic) was performed using reagents from BD Pharmingen ( San Diego, CA) that was followed by the flow cytometry on FACS Calibur flow cytometer (Becton Dickinson) using the CellQuest program. Additionally, Trypan blue exclusion test was used for determination of cell viability and total death levels. Clonogenic survival assay of glioblastoma cells before and after treatment with increased doses of CBD and γ-radiation in the presence or absence of a PI3K-AKT inhibitor LY294002 (40 µM), an IKK-NF-κB inhibitor BMS345541 (10 µM) or several other small molecule inhibitors was also performed using a standard method.

### Western blot analysis

Total cell lysates (50 µg protein) were resolved on SDS-PAGE, and processed according to standard protocols. The antibodies used for Western blotting included anti-β-Actin mouse mAb (Sigma, St. Louis, MO, USA).The antibodies to human antigens obtained from Cell Signaling (Danvers, MA) included phospho-p44/p42 MAPK (Erk1/2) (T202/Y204) rabbit mAb #4377; p44/p42 MAPK (Erk1/2) Ab #9102; phospho-SAPK/JNK (Thr183/Tyr185) rabbit mAb #4668; SAPK/JNK Ab #9252; phospho-cJUN (Ser73) Ab#9164; cJUN mouse mAb #2315; phospho-p38 MAPK (Thr180/Tyr182) rabbit mAb #4511; phospho-ATF2 (Thr71) Ab #9221; ATF2 rabbit mAb #9226; phospho-AKT (Ser473) #927; AKT Ab #9272; phospho-NF-κB p65 (Ser568) rabbit mAb #3033; NF-κB p65 rabbit mAb #4764; phospho-STAT3 (Tyr705) Ab #9131; STAT3 rabbit mAb #4904; p53 Ab #9282; BAX Ab #2772; PARP Ab #9542 and SOX2 Ab (#2748). The antibodies obtained from Cayman (Ann Arbor, MI) included CB1 receptor Ab #101500 and CB2 receptor Ab #101550. The secondary antibodies were conjugated to horseradish peroxidase; signals were detected using the ECL system (Thermo Scientific, Rockford, IL, USA).

### ELISA for TNFα, TRAIL, IL6, IL8 and TGFβ1 detection in the media

The ELISA kits for detection of human cytokines were from R&D System, Minneapolis, MN, USA and eBioscience, San Diego, CA, USA.

### Luciferase reporter assay

TNF-promoter-luciferase reporter activity was determined in transiently transfected glioblastoma cells using-615TNFpr-Luc (wild type), two mutated constructs, -615TNFpr(mutCRE)-Luc and -615TNFpr(mutAP1)-Luc, and -36TNFpr-Luc with “minimal” promoter, as previously described [64, 65] with small modifications. 1.5 kb-TRAILpr-Luc reporter construct was also previously described [66]. Transient transfection of glioblastoma cells was performed using Lipofectamine (Thermo Fisher Scientific). Luciferase activity was measured using a Dual-Luciferase Reporter Assay (Promega). Firefly luciferase activity of TNFpr or TRAILpr reporters was normalized to Renilla luciferase activity. Transfected cells were treated with 0.1% DMSO or 20 µM CBD for 6–18 hours.

### Expression dominant-negative constructs

Dominant-negative constructs pcDNA3-p38-ASP [97] and pcDNA3-JNK1-APF [98] used for regulation of apoptosis by in transiently transfected glioblastoma cells were also used in our previous studies [53, 54]. Cotransfection of glioblastoma cells by indicated expression constructs was performed in the presence of pcDNA3-GFP (5:1) using standard operating Lipofectamine procedure (Thermo Fisher Scientific).

### RNA interference

U87MG cells were seeded at 100000 cells per well into six-well plates and grown for 24 h. Transfection of siRNA (50 nM) was performed using Lipofectamine 2000 (Thermo Fisher Scientific). SignalSilence p38 MAPK siRNA (#6564) and SignalSilence control siRNA (#6568) from “Cell Signaling Technology” were used. Control siRNA (Fluorescein conjugate) (#6201) was added to both preparations (at ratio 1:5). 48 h after transfection, cells were treated by CBD for 6 h (for Western blot analysis) or 24 h for cell-cycle apoptosis analysis using PI staining of fixed cells and the subsequent FACS analysis of green transfected cells.

### Quantitative real-time PCR

U87 and U118 cells were plated in 6-well plates and treated with CBD and/or radiation for indicated time. Cells were harvested using Trizol (Thermo Fisher Scientific) after washing twice with ice-cold PBS. RNA was extracted and converted to cDNA using High Capacity cDNA reverse transcription kit (Thermo Fisher Scientific). The QPCR probe sequences are listed in Table 1.

**Table 1:** QPCR primer sequences

Target Gene	Forward Primer	Reverse Primer
*IL1-B*	ACGCTCCGGGACTCACAGCA	TGAGGCCCAAGGCCACAGGT
*IL6*	TCCACAAGCGCCTTCGGTCC	GTGGCTGTCTGTGTGGGGCG
*IL8*	GGCCGTGGCTCTCTTGGCAG	TGTGTTGGCGCAGTGTGGTCC
*TGFbeta*	CTGCTGGCACCCAGCGACTC	GCAGTGGGCGCTAAGGCGAA
*TNFalpha*	GGCTCCAGGCGGTGCTTGTT	TGACTGCCTGGGCCAGAGGG

Gene expressions were measured by Life Technologies ViiA 7 Real-Time PCR System in standard mode. QPCR was analyzed using the comparative CT method (*ΔΔCT* Method). Beta-actin was used as reference gene. For each sample, the CT value of target gene was first normalized to the beta-actin CT value to obtain ΔCT. Then the ΔCT value was normalized to the control treatment (ctrl) within the same time-point to calculate ΔΔCT value. Showing in the graph is a 2^-ΔΔCT^ value, which indicates the fold change of target gene mRNA levels against time-point control after normalization to reference gene.

### Suppression of cell signaling pathways by specific inhibitors

We performed specific inhibition of several signaling pathways using JNK1-3 inhibitor SP600125 (20 µM), MAPK p38 inhibitor SB203580 (20 µM), IKK-NF-κB inhibitor BMS345541 (10 µM); PI3K-AKT inhibitor LY294002 (50 µM); and MEK-ERK inhibitor U0126 (10 µM). Western blotting with Abs to active forms of targeted proteins was used to evaluate the efficacy of inhibition. We assessed changes in apoptosis levels under these conditions. All inhibitors were dissolved in DMSO. 0.1% or 0.2% DMSO was used as a control vehicle.

### Statistical analyses of data

Data from four to five independent experiments were calculated as means and standard deviations. Comparisons of results between treated and control groups were made by the Students’ *t*-tests. A *p*-value of 0.05 or less between groups was considered significant.

## Abbreviations

AP1: activator protein 1
ATF2: activating transcription factor 2
CHX: cycloheximide
COX2: cyclooxygenase-2
ERK: extracellular signal-regulated kinase
FACS: fluorescence-activated cell sorter
FasL: Fas Ligand
IκB: inhibitor of NF-κB
IKK: inhibitor nuclear factor kappa B kinase
JNK: Jun N-terminal kinase
mAb: monoclonal Ab
Luc: luciferase;
MAPK: mitogen-activated protein kinase
MEK: MAPK kinase
MEKK1: MAPK/ERK kinase: kinase-1
MFI: medium fluorescence intensity
NF-κB: nuclear factor kappa B
PARP: poly(ADP-ribose) polymerase
PI: propidium iodide
PI3K: phosphatidylinositol 3-kinase
ROS: reactive oxygen species
RNAi: RNA interference
STAT: signal transducer and activator of transcription
TNFa: tumor necrosis factor alpha
TNFR: tumor necrosis factor receptor
TRAIL: TNF-related apoptosis-inducing ligand
TRAIL-R: TRAIL-receptor
zVAD-fmk: benzyloxycarbonyl-Val-Ala-Asp-fluoromethyl ketone

## References

[1] Shah BK, Bista A, Sharma S (2016). Survival Trends in Elderly Patients with Glioblastoma in the United States: a Population-based Study. Anticancer Res.

[2] Hottinger AF, Pacheco P, Stupp R (2016). Tumor treating fields: a novel treatment modality and its use in brain tumors. Neuro-oncol.

[3] Monje ML, Mizumatsu S, Fike JR, Palmer TD (2002). Irradiation induces neural precursor-cell dysfunction. Nat Med.

[4] Mizumatsu S, Monje ML, Morhardt DR, Rola R, Palmer TD, Fike JR (2003). Extreme sensitivity of adult neurogenesis to low doses of X-irradiation. Cancer Res.

[5] Acharya MM, Lan ML, Kan VH, Patel NH, Giedzinski E, Tseng BP, Limoli CL (2010). Consequences of ionizing radiation-induced damage in human neural stem cells. Free Radic Biol Med.

[6] Acharya MM, Christie LA, Lan ML, Giedzinski E, Fike JR, Rosi S, Limoli CL (2011). Human neural stem cell transplantation ameliorates radiation-induced cognitive dysfunction. Cancer Res.

[7] Hellström NA, Björk-Eriksson T, Blomgren K, Kuhn HG (2009). Differential recovery of neural stem cells in the subventricular zone and dentate gyrus after ionizing radiation. Stem Cells.

[8] Greene-Schloesser D, Robbins ME, Peiffer AM, Shaw EG, Wheeler KT, Chan MD (2012). Radiation-induced brain injury: A review. Front Oncol.

[9] Brennan CW, Verhaak RG, McKenna A, Campos B, Noushmehr H, Salama SR, Zheng S, Chakravarty D, Sanborn JZ, Berman SH, Beroukhim R, Bernard B, Wu CJ (2013). TCGA Research Network. The somatic genomic landscape of glioblastoma. Cell.

[10] Ivanov VN, Hei TK (2014). A role for TRAIL/TRAIL-R2 in radiation-induced apoptosis and radiation-induced bystander response of human neural stem cells. Apoptosis.

[11] Porter KR, McCarthy BJ, Freels S, Kim Y, Davis FG (2010). Prevalence estimates for primary brain tumors in the United States by age, gender, behavior, and histology. Neuro-oncol.

[12] Louis DN (2006). Molecular pathology of malignant gliomas. Annu Rev Pathol.

[13] Cancer Genome Atlas Research Network (2008). Comprehensive genomic characterization defines human glioblastoma genes and core pathways. Nature.

[14] Chen J, McKay RM, Parada LF (2012). Malignant glioma: lessons from genomics, mouse models, and stem cells. Cell.

[15] Bredel M, Scholtens DM, Yadav AK, Alvarez AA, Renfrow JJ, Chandler JP, Yu IL, Carro MS, Dai F, Tagge MJ, Ferrarese R, Bredel C, Phillips HS (2011). NFKBIA deletion in glioblastomas. N Engl J Med.

[16] Wei W, Shin YS, Xue M, Matsutani T, Masui K, Yang H, Ikegami S, Gu Y, Herrmann K, Johnson D, Ding X, Hwang K, Kim J (2016). Single-Cell Phosphoproteomics Resolves Adaptive Signaling Dynamics and Informs Targeted Combination Therapy in Glioblastoma. Cancer Cell.

[17] Okada H, Mak TW (2004). Pathways of apoptotic and non-apoptotic death in tumour cells. Nat Rev Cancer.

[18] Hitomi J, Christofferson DE, Ng A, Yao J, Degterev A, Xavier RJ, Yuan J (2008). Identification of a molecular signaling network that regulates a cellular necrotic cell death pathway. Cell.

[19] Persano L, Rampazzo E, Basso G, Viola G (2013). Glioblastoma cancer stem cells: role of the microenvironment and therapeutic targeting. Biochem Pharmacol.

[20] Hei TK, Zhou H, Chai Y, Ponnaiya B, Ivanov VN (2011). Radiation induced non-targeted response: mechanism and potential clinical implications. Curr Mol Pharmacol.

[21] Prise KM, O’Sullivan JM (2009). Radiation-induced bystander signalling in cancer therapy. Nat Rev Cancer.

[22] Ivanov VN, Hei TK (2014). Radiation-induced glioblastoma signaling cascade regulates viability, apoptosis and differentiation of neural stem cells (NSC). Apoptosis.

[23] Morgan WF (2003). Non-targeted and delayed effects of exposure to ionizing radiation: II. Radiation-induced genomic instability and bystander effects *in vivo*, clastogenic factors and transgenerational effects. Radiat Res.

[24] Verhaak RG, Hoadley KA, Purdom E, Wang V, Qi Y, Wilkerson MD, Miller CR, Ding L, Golub T, Mesirov JP, Alexe G, Lawrence M, O’Kelly M (2010). Cancer Genome Atlas Research Network. Integrated genomic analysis identifies clinically relevant subtypes of glioblastoma characterized by abnormalities in PDGFRA, IDH1, EGFR, and NF1. Cancer Cell.

[25] Li A, Walling J, Kotliarov Y, Center A, Steed ME, Ahn SJ, Rosenblum M, Mikkelsen T, Zenklusen JC, Fine HA (2008). Genomic changes and gene expression profiles reveal that established glioma cell lines are poorly representative of primary human gliomas. Mol Cancer Res.

[26] Solinas M, Massi P, Cinquina V, Valenti M, Bolognini D, Gariboldi M, Monti E, Rubino T, Parolaro D (2013). Cannabidiol, a non-psychoactive cannabinoid compound, inhibits proliferation and invasion in U87-MG and T98G glioma cells through a multitarget effect. PLoS One.

[27] Iorio F, Knijnenburg TA, Vis DJ, Bignell GR, Menden MP, Schubert M, Aben N, Gonçalves E, Barthorpe S, Lightfoot H, Cokelaer T, Greninger P, van Dyk E (2016). A Landscape of Pharmacogenomic Interactions in Cancer. Cell.

[28] Brown I, Cascio MG, Rotondo D, Pertwee RG, Heys SD, Wahle KW (2013). Cannabinoids and omega-3/6 endocannabinoids as cell death and anticancer modulators. Prog Lipid Res.

[29] Scott KA, Dalgleish AG, Liu WM (2014). The combination of cannabidiol and Δ9-tetrahydrocannabinol enhances the anticancer effects of radiation in an orthotopic murine glioma model. Mol Cancer Ther.

[30] Massi P, Solinas M, Cinquina V, Parolaro D (2013). Cannabidiol as potential anticancer drug. Br J Clin Pharmacol.

[31] Velasco G, Galve-Roperh I, Sánchez C, Blázquez C, Guzmán M (2004). Hypothesis: cannabinoid therapy for the treatment of gliomas?. Neuropharmacology.

[32] Velasco G, Hernández-Tiedra S, Dávila D, Lorente M (2016). The use of cannabinoids as anticancer agents. Prog Neuropsychopharmacol Biol Psychiatry.

[33] Chakravarti B, Ravi J, Ganju RK (2014). Cannabinoids as therapeutic agents in cancer: current status and future implications. Oncotarget.

[34] Maccarrone M, Guzmán M, Mackie K, Doherty P, Harkany T (2014). Programming of neural cells by (endo)cannabinoids: from physiological rules to emerging therapies. Nat Rev Neurosci.

[35] Scotter EL, Abood ME, Glass M (2010). The endocannabinoid system as a target for the treatment of neurodegenerative disease. Br J Pharmacol.

[36] Pertwee RG (2009). Emerging strategies for exploiting cannabinoid receptor agonists as medicines. Br J Pharmacol.

[37] McPartland JM, Duncan M, Di Marzo V, Pertwee RG (2015). Are cannabidiol and Δ(9) -tetrahydrocannabivarin negative modulators of the endocannabinoid system? A systematic review. Br J Pharmacol.

[38] Carracedo A, Lorente M, Egia A, Blázquez C, García S, Giroux V, Malicet C, Villuendas R, Gironella M, González-Feria L, Piris MA, Iovanna JL, Guzmán M, Velasco G (2006). The stress-regulated protein p8 mediates cannabinoid-induced apoptosis of tumor cells. Cancer Cell.

[39] Salazar M, Carracedo A, Salanueva IJ, Hernández-Tiedra S, Lorente M, Egia A, Vázquez P, Blázquez C, Torres S, García S, Nowak J, Fimia GM, Piacentini M (2009). Cannabinoid action induces autophagy-mediated cell death through stimulation of ER stress in human glioma cells. J Clin Invest.

[40] Singer E, Judkins J, Salomonis N, Matlaf L, Soteropoulos P, McAllister S, Soroceanu L (2015). Reactive oxygen species-mediated therapeutic response and resistance in glioblastoma. Cell Death Dis.

[41] Shrivastava A, Kuzontkoski PM, Groopman JE, Prasad A (2011). Cannabidiol induces programmed cell death in breast cancer cells by coordinating the cross-talk between apoptosis and autophagy. Mol Cancer Ther.

[42] Massi P, Valenti M, Solinas M, Parolaro D (2010). Molecular mechanisms involved in the antitumor activity of cannabinoids on gliomas: role for oxidative stress. Cancers (Basel).

[43] De Petrocellis L, Ligresti A, Schiano Moriello A, Iappelli M, Verde R, Stott CG, Cristino L, Orlando P, Di Marzo V (2013). Non-THC cannabinoids inhibit prostate carcinoma growth *in vitro* and *in vivo*: pro-apoptotic effects and underlying mechanisms. Br J Pharmacol.

[44] Velasco G, Sánchez C, Guzmán M (2012). Towards the use of cannabinoids as antitumour agents. Nat Rev Cancer.

[45] Eriksson D, Stigbrand T (2010). Radiation-induced cell death mechanisms. Tumour Biol.

[46] Villalonga-Planells R, Coll-Mulet L, Martínez-Soler F, Castaño E, Acebes JJ, Giménez-Bonafé P, Gil J, Tortosa A (2011). Activation of p53 by nutlin-3a induces apoptosis and cellular senescence in human glioblastoma multiforme. PLoS One.

[47] Marcu JP, Christian RT, Lau D, Zielinski AJ, Horowitz MP, Lee J, Pakdel A, Allison J, Limbad C, Moore DH, Yount GL, Desprez PY, McAllister SD (2010). Cannabidiol enhances the inhibitory effects of delta9-tetrahydrocannabinol on human glioblastoma cell proliferation and survival. Mol Cancer Ther.

[48] Massi P, Vaccani A, Ceruti S, Colombo A, Abbracchio MP, Parolaro D (2004). Antitumor effects of cannabidiol, a nonpsychoactive cannabinoid, on human glioma cell lines. J Pharmacol Exp Ther.

[49] Zhu VF, Yang J, Lebrun DG, Li M (2012). Understanding the role of cytokines in Glioblastoma Multiforme pathogenesis. Cancer Lett.

[50] Degterev A, Hitomi J, Germscheid M, Ch’en IL, Korkina O, Teng X, Abbott D, Cuny GD, Yuan C, Wagner G, Hedrick SM, Gerber SA, Lugovskoy A, Yuan J (2008). Identification of RIP1 kinase as a specific cellular target of necrostatins. Nat Chem Biol.

[51] Wagner EF, Nebreda AR (2009). Signal integration by JNK and p38 MAPK pathways in cancer development. Nat Rev Cancer.

[52] Kaminskyy VO, Zhivotovsky B (2014). Free radicals in cross talk between autophagy and apoptosis. Antioxid Redox Signal.

[53] Ivanov VN, Fodstad O, Ronai Z (2001). Expression of ring finger-deleted TRAF2 sensitizes metastatic melanoma cells to apoptosis via up-regulation of p38, TNFalpha and suppression of NF-kappaB activities. Oncogene.

[54] Ivanov VN, Ronai Z (2000). p38 protects human melanoma cells from UV-induced apoptosis through down-regulation of NF-kappaB activity and Fas expression. Oncogene.

[55] Karin M (2006). Nuclear factor-kappaB in cancer development and progression. Nature.

[56] Karin M, Greten FR (2005). NF-kappaB: linking inflammation and immunity to cancer development and progression. Nat Rev Immunol.

[57] Mattson MP, Meffert MK (2006). Roles for NF-kappaB in nerve cell survival, plasticity, and disease. Cell Death Differ.

[58] Karin M, Cao Y, Greten FR, Li ZW (2002). NF-kappaB in cancer: from innocent bystander to major culprit. Nat Rev Cancer.

[59] Joki T, Heese O, Nikas DC, Bello L, Zhang J, Kraeft SK, Seyfried NT, Abe T, Chen LB, Carroll RS, Black PM (2000). Expression of cyclooxygenase 2 (COX-2) in human glioma and *in vitro* inhibition by a specific COX-2 inhibitor, NS-398. Cancer Res.

[60] Karin M (2005). Inflammation-activated protein kinases as targets for drug development. Proc Am Thorac Soc.

[61] Sabio G, Davis RJ (2014). TNF and MAP kinase signalling pathways. Semin Immunol.

[62] Faris M, Latinis KM, Kempiak SJ, Koretzky GA, Nel A (1998). Stress-induced Fas ligand expression in T cells is mediated through a MEK kinase 1-regulated response element in the Fas ligand promoter. Mol Cell Biol.

[63] Holtz-Heppelmann CJ, Algeciras A, Badley AD, Paya CV (1998). Transcriptional regulation of the human FasL promoter-enhancer region. J Biol Chem.

[64] Rhoades KL, Golub SH, Economou JS (1992). The regulation of the human tumor necrosis factor alpha promoter region in macrophage, T cell, and B cell lines. J Biol Chem.

[65] Ivanov VN, Ronai Z (1999). Down-regulation of tumor necrosis factor alpha expression by activating transcription factor 2 increases UVC-induced apoptosis of late-stage melanoma cells. J Biol Chem.

[66] Baetu TM, Kwon H, Sharma S, Grandvaux N, Hiscott J (2001). Disruption of NF-kappaB signaling reveals a novel role for NF-kappaB in the regulation of TNF-related apoptosis-inducing ligand expression. J Immunol.

[67] Herzer K, Grosse-Wilde A, Krammer PH, Galle PR, Kanzler S (2008). Transforming growth factor-beta-mediated tumor necrosis factor-related apoptosis-inducing ligand expression and apoptosis in hepatoma cells requires functional cooperation between Smad proteins and activator protein-1. Mol Cancer Res.

[68] Herr I, Posovszky C, Di Marzio LD, Cifone MG, Boehler T, Debatin KM (2000). Autoamplification of apoptosis following ligation of CD95-L, TRAIL and TNF-alpha. Oncogene.

[69] Allen JE, El-Deiry WS (2012). Regulation of the human TRAIL gene. Cancer Biol Ther.

[70] Opel D, Westhoff MA, Bender A, Braun V, Debatin KM, Fulda S (2008). Phosphatidylinositol 3-kinase inhibition broadly sensitizes glioblastoma cells to death receptor- and drug-induced apoptosis. Cancer Res.

[71] Beg AA, Sha WC, Bronson RT, Ghosh S, Baltimore D (1995). Embryonic lethality and liver degeneration in mice lacking the RelA component of NF-kappa B. Nature.

[72] Luo JL, Kamata H, Karin M 1 (2005). The anti-death machinery in IKK/NF-kappaB signaling. J Clin Immunol.

[73] Bagó JR, Alfonso-Pecchio A, Okolie O, Dumitru R, Rinkenbaugh A, Baldwin AS, Miller CR, Magness ST, Hingtgen SD (2016). Therapeutically engineered induced neural stem cells are tumour-homing and inhibit progression of glioblastoma. Nat Commun.

[74] Li C, Wang XR, Tang YD, An Y, Zhou YS, Guo SW, Zhang XY, Duan TJ, Zhu JX, Li XF, Wang LZ, Wang CH, Wang YF (2012). [A multicenter study of coronary artery disease and its risk factors in rheumatoid arthritis in China]. [Article in Chinese]. Beijing Da Xue Xue Bao.

[75] Massi P, Vaccani A, Bianchessi S, Costa B, Macchi P, Parolaro D (2006). The non-psychoactive cannabidiol triggers caspase activation and oxidative stress in human glioma cells. Cell Mol Life Sci.

[76] McAllister SD, Soroceanu L, Desprez PY (2015). The Antitumor Activity of Plant-Derived Non-Psychoactive Cannabinoids. J Neuroimmune Pharmacol.

[77] Pertwee RG (2008). The diverse CB1 and CB2 receptor pharmacology of three plant cannabinoids: delta9-tetrahydrocannabinol, cannabidiol and delta9-tetrahydrocannabivarin. Br J Pharmacol.

[78] Wolchok JD, Kluger H, Callahan MK, Postow MA, Rizvi NA, Lesokhin AM, Segal NH, Ariyan CE, Gordon RA, Reed K, Burke MM, Caldwell A, Kronenberg SA (2013). Nivolumab plus ipilimumab in advanced melanoma. N Engl J Med.

[79] Galluzzi L, Lugli E (2013). Cancer immunotherapy turns viral. OncoImmunology.

[80] Araki K, Youngblood B, Ahmed R (2013). Programmed cell death 1-directed immunotherapy for enhancing T-cell function. Cold Spring Harb Symp Quant Biol.

[81] Vara D, Salazar M, Olea-Herrero N, Guzmán M, Velasco G, Díaz-Laviada I (2011). Anti-tumoral action of cannabinoids on hepatocellular carcinoma: role of AMPK-dependent activation of autophagy. Cell Death Differ.

[82] Kang R, Zeh HJ, Lotze MT, Tang D (2011). The Beclin 1 network regulates autophagy and apoptosis. Cell Death Differ.

[83] Karin M (2009). NF-kappaB as a critical link between inflammation and cancer. Cold Spring Harb Perspect Biol.

[84] Vivanco I, Sawyers CL (2002). The phosphatidylinositol 3-Kinase AKT pathway in human cancer. Nat Rev Cancer.

[85] Ströbele S, Schneider M, Schneele L, Siegelin MD, Nonnenmacher L, Zhou S, Karpel-Massler G, Westhoff MA, Halatsch ME, Debatin KM (2015). A Potential Role for the Inhibition of PI3K Signaling in Glioblastoma Therapy. PLoS One.

[86] Zanotto-Filho A, Braganhol E, Schröder R, de Souza LH, Dalmolin RJ, Pasquali MA, Gelain DP, Battastini AM, Moreira JC (2011). NFκB inhibitors induce cell death in glioblastomas. Biochem Pharmacol.

[87] Shiizaki S, Naguro I, Ichijo H (2013). Activation mechanisms of ASK1 in response to various stresses and its significance in intracellular signaling. Adv Biol Regul.

[88] Maruyama T, Araki T, Kawarazaki Y, Naguro I, Heynen S, Aza-Blanc P, Ronai Z, Matsuzawa A, Ichijo H (2014). Roquin-2 promotes ubiquitin-mediated degradation of ASK1 to regulate stress responses. Sci Signal.

[89] Allen JE, Krigsfeld G, Mayes PA, Patel L, Dicker DT, Patel AS, Dolloff NG, Messaris E, Scata KA, Wang W, Zhou JY, Wu GS, El-Deiry WS (2013). Dual inactivation of Akt and ERK by TIC10 signals Foxo3a nuclear translocation, TRAIL gene induction, and potent antitumor effects. Sci Transl Med.

[90] Kline CL, Van den Heuvel AP, Allen JE, Prabhu VV, Dicker DT, El-Deiry WS (2016). ONC201 kills solid tumor cells by triggering an integrated stress response dependent on ATF4 activation by specific eIF2α kinases. Sci Signal.

[91] Greenhough A, Smartt HJ, Moore AE, Roberts HR, Williams AC, Paraskeva C, Kaidi A (2009). The COX-2/PGE2 pathway: key roles in the hallmarks of cancer and adaptation to the tumour microenvironment. Carcinogenesis.

[92] Hermanson DJ, Hartley ND, Gamble-George J, Brown N, Shonesy BC, Kingsley PJ, Colbran RJ, Reese J, Marnett LJ, Patel S (2013). Substrate-selective COX-2 inhibition decreases anxiety via endocannabinoid activation. Nat Neurosci.

[93] Borges HL, Linden R, Wang JY (2008). DNA damage-induced cell death: lessons from the central nervous system. Cell Res.

[94] Clark MJ, Homer N, O’Connor BD, Chen Z, Eskin A, Lee H, Merriman B, Nelson SF (2010). U87MG decoded: the genomic sequence of a cytogenetically aberrant human cancer cell line. PLoS Genet.

[95] Allen M, Bjerke M, Edlund H, Nelander S, Westermark B (2016). Origin of the U87MG glioma cell line: good news and bad news. Sci Transl Med.

[96] Ivanov VN, Hei TK (2013). Induction of apoptotic death and retardation of neuronal differentiation of human neural stem cells by sodium arsenite treatment. Exp Cell Res.

[97] Enslen H, Raingeaud J, Davis RJ (1998). Selective activation of p38 mitogen-activated protein (MAP) kinase isoforms by the MAP kinase kinases MKK3 and MKK6. J Biol Chem.

[98] Butterfield L, Storey B, Maas L, Heasley LE (1997). c-Jun NH2-terminal kinase regulation of the apoptotic response of small cell lung cancer cells to ultraviolet radiation. J Biol Chem.

